# Multidrug Resistant *Acinetobacter* Isolates Release Resistance Determinants Through Contact-Dependent Killing and Bacteriophage Lysis

**DOI:** 10.3389/fmicb.2020.01918

**Published:** 2020-08-14

**Authors:** Clay S. Crippen, Michael J. Rothrock Jr., Susan Sanchez, Christine M. Szymanski

**Affiliations:** ^1^Department of Microbiology and Complex Carbohydrate Research Center, University of Georgia, Athens, GA, United States; ^2^United States National Poultry Research Center, Agricultural Research Service, United States Department of Agriculture, Athens, GA, United States; ^3^Athens Veterinary Diagnostic Lab, Department of Infectious Diseases, University of Georgia, Athens, GA, United States

**Keywords:** *Acinetobacter*, environmental isolates, multidrug resistance, contact-dependent killing, bacteriophages, gene transfer

## Abstract

Antimicrobial resistance is an ancient bacterial defense mechanism that has rapidly spread due to the frequent use of antibiotics for disease treatment and livestock growth promotion. We are becoming increasingly aware that pathogens, such as members of the genus *Acinetobacter*, are precipitously evolving drug resistances through multiple mechanisms, including the acquisition of antibiotic resistance genes. In this study, we isolated three multidrug resistant *Acinetobacter* species from birds on a free-range farm. *Acinetobacter radioresistens*, *Acinetobacter lwoffii*, and *Acinetobacter johnsonii* were isolated from hens, turkeys and ducks and were resistant to 14 clinically relevant antibiotics, including several listed by the World Health Organization as essential medicines. Co-culturing any of the three *Acinetobacter* species with *Acinetobacter baumannii* resulted in contact-dependent release of intact resistance determinants. We also isolated several lytic bacteriophages and selected two of these phages to be included in this study based on differences in plaquing characteristics, nucleic acid content and viral morphology. Both phages released host DNA, including antibiotic resistance genes during cell lysis and we demonstrated that these resistance determinants were transferable to a naïve strain of *Escherichia coli*. This study demonstrates that contact-dependent competition between bacterial species can readily contribute to DNA release into the environment, including antibiotic resistance determinants. We also highlight that the constant lysis and turnover of bacterial populations during the natural lifecycle of a lytic bacteriophage is an underappreciated mechanism for the liberation of DNA and subsequent genetic exchange.

## Introduction

In recent years, multidrug resistant (MDR) bacteria have become a serious concern for healthcare providers worldwide (Poirel et al., 2011; Bengtsson-Palme et al., 2017). Preeminent among these bacteria is *Acinetobacter baumannii*, a common nosocomial MDR pathogen resistant to desiccation and readily able to acquire MDR genes in both hospital and environmental settings (Poirel et al., 2008; Al Atrouni et al., 2016). In 2013, the CDC ranked carbapenem-resistant *A. baumannii* as the most concerning MDR pathogen requiring new antibiotics (Sievert et al., 2013) and the WHO published a similar report in 2017 (Lawe-Davies and Bennett, 2017). At the origin of this problem is the propensity of *A. baumannii* to become resistant to antibiotic treatment through acquisition of resistance genes (Wilharm et al., 2013). Although the extreme resistances of clinical isolates of *A. baumannii* are well documented (Valentine et al., 2008; Yang et al., 2015; Fernando et al., 2016), the prevalence of MDR isolates in agricultural settings is only recently being explored (Maboni et al., 2020). Furthermore, there has been limited characterization of *A. baumannii* interactions with reservoir species, such as *Acinetobacter radioresistens*, which can serve as a source of carbapenem resistance in hospital settings (Poirel et al., 2008). Thus, identifying MDR gene reservoirs and modes of gene release to naïve pathogens will be critical to understanding and combating the spread of antibiotic resistance.

In addition to encoding a number of antibiotic resistance determinants, *A. baumannii* is well equipped to outcompete neighboring bacteria in a number of approaches. The genus *Acinetobacter* has been confirmed to carry type 1 (T1SS), type 2 (T2SS), type 4 (T4SS), and type 6 (T6SS) secretion systems, as well as >40 contact-dependent inhibition (CDI) systems, all of which are virulence factors that give *Acinetobacter* species a competitive advantage (Weber et al., 2013; Hayes et al., 2014; Souza et al., 2015; Harding et al., 2017; Kinsella et al., 2017; De Gregorio et al., 2019; Sgro et al., 2019). T6SS and CDI systems are two mechanisms of direct bacterial competition that *Acinetobacter* species encode (Harding et al., 2018). Briefly, the T6SS is a complex multi-component system anchored in the inner cell membrane that builds a spike, which, upon contact, injects toxins into a prey cell (Weber et al., 2013). CDI is a two-component system anchored in the outer membrane, which consists of a large transporter (CdiB) and toxin (CdiA) that is released during contact with the prey cell (De Gregorio et al., 2018; Roussin et al., 2019). These virulence factors greatly increase the fitness of the acinetobacters that express them and enables the bacteria to thrive in diverse and competitive environments.

In recent years, there has been an effort to isolate more bacteriophages that use ESKAPE pathogens, such as *A. baumannii*, as a host. Bacteriophages have been and still are an under-appreciated distributor of antimicrobial resistance elements. These microbial viruses, which have been reported to infect ∼10^24^ bacterial cells per second globally (Deresinski, 2009), are the most abundant biological entity on earth (Suttle, 2005; Paez-Espino et al., 2016). Host gene transduction, including MDR genes, has been described for some bacteriophages and phage-derived elements (Penades et al., 2015). Although the mechanisms are poorly understood, it was generally accepted that upon infection, most non-transducing lytic bacteriophages will degrade host DNA to inhibit cellular activities or to repurpose nucleotides for viral use (Warren and Bose, 1968). However, there have been recent observations that some lytic phages release intact plasmid DNA (Keen et al., 2017). Keen et al. (2017) have designated these phages as “superspreaders” and demonstrated that drug resistance genes released by these phages can be acquired and expressed by unrelated bacteria found in the same environment. Although over 100 *Acinetobacter-*infecting bacteriophages have been isolated, this phenomenon has not been reported for this genus (Turner et al., 2017). A greater understanding of *Acinetobacter* phage impact on MDR dissemination is required before designing phage therapies to treat antibiotic resistant infections, except in the most desperate cases.

Understanding how *Acinetobacter* species are exposed to exogenous genetic material is crucial to further understanding MDR spread. In this report, we describe MDR *Acinetobacter* species isolated from a free-range farm (Rothrock et al., 2016). Based on their susceptibility to phage predation and killing when co-cultured with *A. baumannii*, we demonstrate that antibiotic resistance genes can be released by contact- and phage-lysed cells. In addition to identifying a potential reservoir of MDR-carrying *Acinetobacter* species in food animals, these findings highlight two underappreciated modes of DNA release that can partially account for the unchecked spread of antibiotic resistance within this genus.

## Materials and Methods

### Strain Isolation and Growth

Strains were isolated from fecal samples collected from layer hens, turkeys, and ducks on a free-range pastured poultry farm (Rothrock et al., 2016). Fecal samples were diluted 2 mL/g in sterile phosphate-buffered saline and plated on Brilliance Campycount agar (Remel) under microaerobic conditions (10% CO_2_, 5% O_2_, 85% N_2_) at 37°C for 18 h. Isolated red colonies were re-cultured on Campy-Line selective agar (Line, 2001).

### Strain Identification

Cell suspensions from agar plates were adjusted to an OD_600_ = 0.5. Suspensions were floated on formvar-coated copper grids for 1 h and 5% paraformaldehyde (Electron Microscopy Sciences, Hatfield, PA, United States) was added to fix the samples before imaging. Transmission electron microscopy (TEM) was performed using a JEOL JEM1011 microscope (JEOL Inc., Peabody, MA, United States). MALDI-TOF VITEK^TM^ MS (BioMérieux, Durham, NC, United States), and, when necessary, full 16S rRNA and *rpoB* sequencing (La Scola et al., 2006) confirmed the isolate identities with 99.9% confidence.

### *Acinetobacter* Growth Conditions

After positive *Acinetobacter* identification, the three *Acinetobacter* species we isolated: *A. radioresistens*, *A. johnsonii*, and *A. lwoffii*, along with the laboratory strains of *A. baumannii*, were incubated aerobically using Luria-Bertani (LB) medium (Becton, Dickinson and Company) at 30°C and under agitation at 200 RPM for liquid cultures.

### Antibiotic Minimum Inhibitory Concentration (MIC) Testing

Antibiotic MIC values for all *Acinetobacter* isolates was assessed using the Vitek 2XL (BioMérieux) and Sensititre (Trek Diagnostic Systems, Cleveland, OH, United States) platforms. The cards/plates used where GN65, GN69 (BioMérieux) and TrekCOMPGN1F, TrekGN4GF (Trek Diagnostic Systems, West Sussex, United Kingdom), following the instructions of the manufacturers and the CLSI guidelines (CLSI M07-A10). Two different MIC systems were used since some isolates did not grow in the Vitek2 card system. Antibiotic break points for both MIC methods were determined by CLSI M100 or EUCAST^1^ when no CLSI break points were published. Isolates were considered MDR if they were resistant to ≥3 antibiotic classes (Manchanda et al., 2010).

### Antibiotic Gradient Diffusion Testing

Imipenem resistance for *A. radioresistens* was also assessed using the imipenem ETEST^®^ strip (BioMérieux). Briefly, a culture of *A. radioresistens* LH6 was adjusted to an OD_600_ = 0.7 in Mueller-Hinton (MH) broth and 150 μL was spread onto MH agar plates. The *E*-test strip was placed on top of the plate and incubated at 37°C overnight prior to imaging on the Chemidoc XRS+ imager (Bio-Rad).

### Plasmid Introduction Into *Acinetobacter* Isolates

The pBAV1K-T5-*gfp* plasmid (Bryksin and Matsumura, 2010) was introduced into LH2, LH6, and D16 by washing overnight liquid cultures with ice-cold 10% glycerol 3X. Then, 50 μL of the cell preparation was mixed with 500 ng of plasmid DNA in an electrocuvette and electroporation was done using the Micropulser Electroporation Apparatus (Bio-Rad) on the Ec2 (2.5 kV, 100 Ω and 25 μF) bacterial setting. The sample was mixed with 100 μL SOC medium [2% tryptone (w/v), 0.5% yeast extract (w/v), 10 mM NaCl, 2.5 mM KCl, 10 mM MgCl_2_, 20 mM glucose] and plated on LB-kanamycin (50 mg/mL) selective plates to isolate plasmid-containing colonies.

### Bacterial Killing Assay

The contact-dependent killing assay was adapted from a previously described bacterial competition assay (Weber et al., 2015). Briefly, strains were grown in liquid culture and adjusted to OD_600_ = 1.0. Strains were washed in LB one time to remove any antibiotic, mixed in a 1:1 ratio and 5 μL was spotted onto LB agar. After 4 h, the agar containing the spot was excised and resuspended into 1 mL of LB broth. Ten-fold serial dilutions were made and spotted onto LB plates supplemented with 50 μg/mL kanamycin (GoldBio) to select for the prey strain and incubated overnight at 30°C to enumerate the surviving strain. When assessing contact-dependent inhibition, a 0.45 μm nitrocellulose membrane (Bio-Rad) separated the strains by spotting the prey strain on LB agar, placing the membrane on the prey strain, and spotting the predator strain on top of the nitrocellulose. This allows the possible passage of small molecules between the predator and prey during the 4-h incubation, but prevents migration of the predator (results not shown). After the incubation time, the membrane and predator strain were removed, and the agar spot was excised and resuspended in PBS for extracellular DNA isolation.

### Extracellular DNA Isolation

Following the co-culture experiments described above, cells were resuspended in phosphate-buffered saline. For all extracellular DNA isolations, cell suspensions and released DNA were filtered through a 0.22 μm membrane. The nucleic acids in the filtrate were isolated via phenol/chloroform (Thermo Fisher Scientific) extraction, precipitated with isopropanol and sodium acetate (Thermo Fisher Scientific), and resuspended in nuclease-free water. Care was taken to ensure that an equal fraction of the aqueous phase was taken from each sample so that the isolated DNA quantities would be proportional to each other.

### PCR Detection of the Kanamycin Resistance Gene

Isolated extracellular DNA was PCR amplified using the kanamycin resistance gene primers: KanF-(CGCAGAAGGCAATGTCATAC) and KanR-(CACTTTGAACGGCATGATGG). Taq polymerase (Thermo Fisher Scientific) was used according to the manufacturer’s instructions, with a *T*_*m*_ of 55°C.

### Bacteriophage Isolation and Propagation

Bacteriophage isolation was performed as described (Gencay et al., 2017). All *Acinetobacter* strains were tested and only LH6 was capable of being infected by all phages. Thus, LH6 was used as the propagating strain and was grown overnight at 30°C with shaking at 200 RPM, the culture was then adjusted to OD_600_ = 0.4, and infected with bacteriophages at a multiplicity of infection (MOI) of 0.0001. The infected culture was incubated at 30°C with shaking at 200 RPM overnight. Afterward, the culture was centrifuged at 4255 × *g* for 15 min, the resulting supernatant was put through a 0.22 μm filter, and the phage-containing filtrate was collected.

### Bacteriophage DNA Release Assay

The *gfp*-plasmid containing strain LH6g (LH6 with *gfp*) was grown in LB liquid culture at 30°C with shaking at 200 RPM overnight. The culture was adjusted to OD_600_ = 1.0, infected with phages at MOI = 0.001, and incubated at 30°C with shaking at 200 RPM for 18 h. DNA isolation was performed as described above.

### Transformation of Released DNA Into Chemically Competent *Escherichia coli* Cells

Transformation of chemically competent TOP10 *E. coli* cells (Invitrogen) was done according to the manufacturer’s instructions. Briefly, 10 μL of isolated released DNA was incubated with 40 μL of competent cells on ice for 15 min. The cells were then heat shocked at 42°C for 45 s and placed back on ice for 2 min. Cells were mixed with 400 μL of LB medium and incubated at 37°C, 200 RPM for 45 min. This was followed by plating the cells on LB plates supplemented with 50 μg/mL kanamycin and incubation overnight at 37°C before counting the transformants.

## Results

### Isolation of MDR *Acinetobacter* Species From Laying Hen, Duck, and Turkey Feces

While isolating campylobacters for our routine studies, colonies typically indicative of *Campylobacter jejuni* or *Campylobacter coli* from Brilliance^TM^ agar followed by sub-culturing on Campy-Line agar were examined by TEM. These isolates did not show the characteristic curved rod morphology associated with *C. jejuni* and *C. coli* (results not shown). To obtain species identification with 99.9% confidence on our isolates, we compared each isolate by MALDI-TOF mass spectrometry, entire 16S rRNA sequencing, and *rpoB* sequencing when necessary (see Supplementary Sequencing Data). We also examined the antibiotic MIC values for these strains since we predicted these isolates would possess multiple resistances allowing them to be cultured on *C. jejuni*/*C. coli* selective plates. Table 1 summarizes the species identification and MIC results. Three *Acinetobacter* species were isolated: *A. radioresistens*, *A. johnsonii*, and *A. lwoffii*. In total, the isolates were resistant to 14 antibiotics, seven of which are on the WHO list of essential medicines (ampicillin, cefazolin, ceftazidime, chloramphenicol, nitrofurantoin, rifampicin, and tetracycline) (WHO, 2017). Additionally, 8/10 isolates were MDR (Manchanda et al., 2010) and were obtained from all bird hosts sampled (laying hen, turkey, and duck). Nine MICs were found to be exclusive to one bird type (laying hen- ceftriaxone, chloramphenicol, rifampicin; turkey- ertapenem, ceftazidime, aztreonam, tetracycline, and cefalexin ticarcillin/clavulanic acid), indicated in gray Table 1. These groupings suggested that drug resistances could be mobile within the respective laying hen and turkey populations, leading us to investigate drug resistance gene mobility further.

**TABLE 1:** Strain identification and minimum inhibitory concentrations of antibiotics for each *Acinetobacter* species isolated from avian fecal samples.

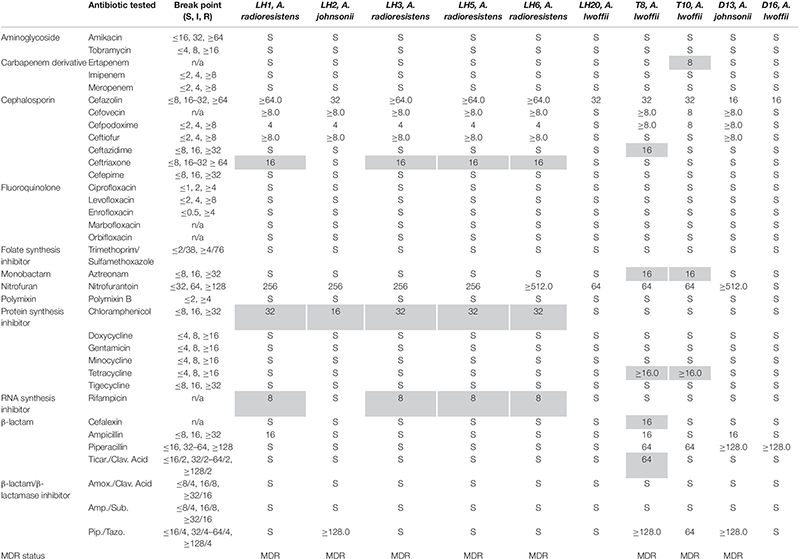

*Identifications were made by MALDI-TOF mass spectroscopy and confirmed by whole 16S rRNA subunit sequencing, and when necessary *rpoB* sequencing. LH, laying hen; T, turkey; D, duck; MIC is reported in units of mg/mL; S, strain was found sensitive to the given antibiotic; Gray boxes indicate which antibiotic resistances are unique to a particular bird host.*

### Characterization of *Acinetobacter* Contact-Dependent Cell Killing

**FIGURE 1:**
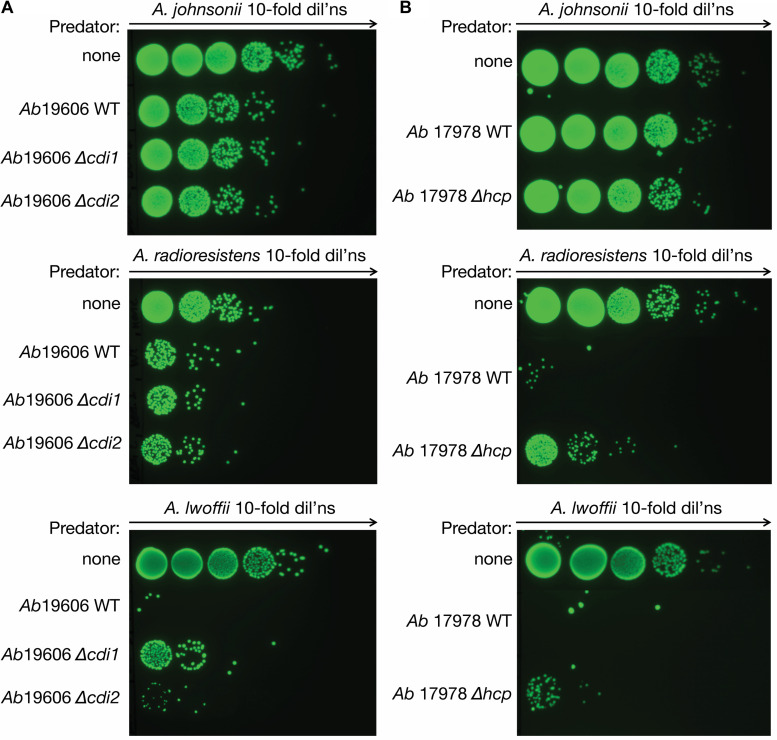
Bacterial killing assay. *Acinetobacter* contact-dependent killing. **(A)** *A. baumannii* kills all isolated *Acinetobacter* species (LH2, LH6, and D16). The surviving isolates are shown after 4 h of contact. A *gfp*-encoding plasmid was introduced into the prey species to distinguish them from *A. baumannii* strain 19606. The *A. baumannii* ATCC 19606 *cdi1* and *cdi2* mutant strains were compared to WT and no competition controls to assess the activity of CDI on the isolated strains. **(B)** The role of T6SS-dependent killing was tested for the isolated strains (LH2, LH6, and D16). The *A. baumannii* strain 17978 *hcp* mutant strain, deficient in T6SS activity, was compared to WT and no competition. The surviving isolates are shown after 4 h of contact. All assays were performed at least in triplicate.

We hypothesized that contact-dependent killing mechanisms present in *Acinetobacter* species could assist in obtaining DNA from neighboring organisms by accelerating the death and lysis of those cells. Therefore, we performed bacterial competition assays to determine whether the isolated *Acinetobacter* species showed a reduction in cell numbers after incubation with *A. baumannii*. The clinically relevant pathogen *A. baumannii* ATCC 19606, which has a constitutively active T6SS, was used as the predator (Weber et al., 2015). After introducing the *gfp*-containing plasmid, pBAV1K-T5-*gfp*, into our *Acinetobacter* prey strains (*A. johnsonii* LH2, *A. radioresistens* LH6, and *A. lwoffii* D16), competition assays were performed to observe the susceptibility of these strains to *A. baumannii* 19606. Killing was observed for all strains, with LH2g (g denotes *gfp*-expressing variant) and D16g showing the most dramatic effect (Figure 1A). Incubating *A. baumannii* 19606 with D16g reduced the prey strains to the limit of detection, while LH2g and LH6g were reduced by ∼100-fold. In an effort to determine if the killing was through general CDI or T6SS, we obtained *cdi1* and *cdi2* deletion mutants in strain 19606 and an *hcp* (hemolysin co-regulated protein) mutant in *A. baumannii* ATCC 17978 (Weber et al., 2015; Harding et al., 2017) and compared their killing relative to the respective WT strains. The *hcp* mutation renders the strain unable to form the extracellular “needle” portion of the T6SS complex, causing the predator to be unable to interact with prey in a T6SS-dependent manner (Ho et al., 2014). We used both strains because we could not generate an *hcp* mutant in strain 19606 and bioinformatic analysis did not identify CDI systems in strain 17978. We observed minimal effect of CDI knockouts in LH2g and LH6g, compared to WT, while the deletion of *cdi1* resulted in greater survival in prey strain D16g, compared to WT and the *cdi2* deletion mutant (Figure 1A). When testing the *hcp* mutant, there was a contribution to killing by the T6SS with LH6g and D16g, and minimal killing of LH2g (Figure 1B). From these results, we concluded that LH2 has minimal susceptibility to CDI and T6SS, LH6 has minimal susceptibility to CDI, but is susceptible to T6SS, and D16 is susceptible to both CDI and T6SS.

### Detection of DNA Released During Contact-Dependent Killing of *Acinetobacter* Isolates

**FIGURE 2:**
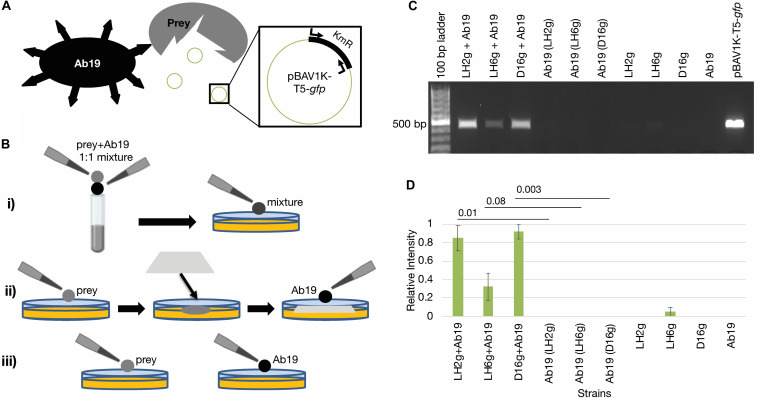
Contact-dependent DNA release. **(A)** Schematic showing assay concept. The predator strain (Ab19) liberates the pBAV1K-T5-*gfp* plasmid from the prey strains being tested. Then the presence of the plasmid is detected by amplification of the KmR gene on the plasmid. **(B)** Schematic detailing the different incubation conditions being used. (i) Prey strain and Ab19 are mixed 1:1 and spotted directly onto the agar plate; (ii) prey strain and Ab19 are separated by a membrane; (iii) controls include incubations of each strain on the plates individually. **(C)** A 1.0% agarose gel depicting the PCR-based KmR gene (450 bp) detection of released DNA mediated by contact dependent killing representative of triplicate experiments shown in Supplementary Figure S1. Co-cultures separated by a membrane to inhibit T6SS are denoted with parentheses. **(D)** Relative intensities of PCR products in each lane from Supplementary Figure S1 were measured by densitometry using Image Lab^TM^. The band produced by amplification of pBAV1K-T5-*gfp* was used as the standard (Rel. Intensity = 1.0). The averages are represented, and error bars represent the standard error of the mean. Student’s paired *T*-tests were performed for each co-incubation compared to its corresponding membrane-separated control incubation. The *p*-values for the LH2g, LH6g, and D16g datasets are indicated.

We next determined if, after co-incubation with *A. baumannii*, the prey strains can release intact DNA into the environment through contact-dependent killing (schematic in Figure 2A). The *A. baumannii* 19606 competition assays were therefore repeated with and without a nitrocellulose membrane between the two strains (to inhibit cell contact, while facilitating the exchange of other molecules) and the DNA was isolated after co-culture (Figure 2B). To test whether or not the released DNA contains intact antibiotic resistance genes, the kanamycin resistance (KmR) gene on the *gfp*-plasmid was probed by PCR. Figure 2C and Supplementary Figure S1 show that the KmR gene was present in all recovered DNA samples from co-cultures, but not in cultures where contact was inhibited by the membrane or in control incubations. The relative intensities of the PCR products in each lane were measured by densitometry, and significantly more plasmid was released by LH2g and D16g during contact than when inhibited by a membrane (Figure 2D). This demonstrates that contact is necessary for cell killing and that an intact KmR gene is released from these cells.

### Detection and Uptake of DNA Released During Bacteriophage-Mediated Cell Killing

**FIGURE 3:**
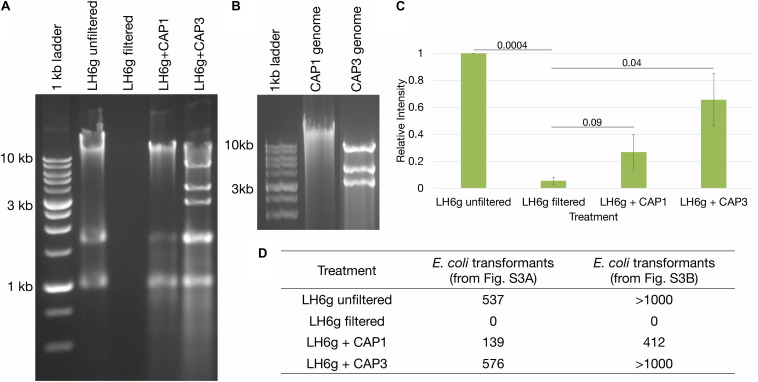
Bacteriophage-mediated DNA release. **(A)** Analyses of extracellular DNA from cultures with and without phage infection. Agarose gel electrophoresis showing intact DNA release from phage-infected cultures, including cellular RNA at approximately 1 and 1.7 kb (see Supplementary Figure S2). **(B)** Agarose gel electrophoresis of CAP1 and CAP3 genomes. **(C)** Relative intensities of PCR products from Supplementary Figure S3 were averaged for each treatment and are represented from triplicate experiments. Standard error of the mean is represented by the error bars, and Student’s paired *T*-tests were performed with *p*-values indicated. **(D)** Chemically competent *E. coli* were transformed with equal amounts of isolated DNA from sample sets from Supplementary Figures S3A,B and the number of kanamycin resistant colonies are indicated.

During initial strain isolation, we also isolated several bacteriophages capable of propagating on *A. radioresistens* strain LH6 with two differing phenotypes: a typical “pinprick” and a “halo” plaque morphology. One representative phage from each phenotypic group (CAP1 and CAP3, respectively) was selected to determine if intact KmR DNA is released after phage infection and cell lysis. The CAP1 phage is a DNA phage likely belonging to the *podoviridae* and the CAP3 phage is an RNA phage belonging to the *cystoviridae*. The characterization of the isolated phages will be described elsewhere (Crippen et al., in preparation). To test the ability of phages CAP1 and CAP3 to release host DNA that contains MDR genes, we performed a phage-mediated DNA release assay. The *gfp*-expressing *A. radioresistens* LH6g was infected with either CAP1 or CAP3 overnight until a clear culture was obtained and the released host DNA was recovered along with encapsidated phage nucleic acids (Figures 3A,B). Bands at approximately 1 and 1.7 kb were determined to be cell RNA (Supplementary Figure S2). PCR amplification with KmR gene primers indicated that both CAP1 and CAP3 released more KmR DNA than was released during cell growth (Figure 3C and Supplementary Figure S3). This shows that these phages exhibit the potential to accelerate the spread of antibiotic resistance genes through the release of intact resistance genes. We further wished to test the transferability of the released DNA by transforming chemically competent *E. coli* cells with equal amounts of released DNA and plating for *gfp*-expressing colonies on Km selective media (Figure 3D). We found that released DNA did contain intact plasmids, which was transformed into *E. coli* cells. Together, these results show that phage-released DNA can be source of resistance determinants for competent cells.

## Discussion

In this study, we unexpectedly isolated three *Acinetobacter* species on *C. jejuni/C. coli* selective media from laying hen, turkey and duck fecal samples obtained from a free-range pasture fed farm. Species such as *C. jejuni* are ubiquitous on poultry farms and have been shown to develop resistances, even in the absence of a selective pressure (Luo et al., 2005; Luangtongkum et al., 2009). Previously, a high incidence of MDR *E. coli* and *Listeria* spp. were also detected in bird fecal samples from this farm, however, *Acinetobacter* species were not examined in that study (Rothrock et al., 2016). The species that we isolated were resistant to 14/31 clinically relevant antibiotics that were tested. Each of these *Acinetobacter* species has been previously reported to carry carbapenemase genes on the *bla*_NDM_ or *bla*_OXA_ containing plasmids, the latter predicted to have actually originated from *A. radioresistens* (Poirel et al., 2008; Zong and Zhang, 2013; Yang et al., 2015). Given that those plasmids are found in divergent *Acinetobacter* isolates, it is unlikely that the genes are subject to exclusive vertical transmission. In this study, 8/10 of our reservoir isolates were MDR, with one *A. lwoffii* MDR isolate showing an additional resistance to the last resort carbapenem, ertapenem, with an MIC of 8.0 mg/mL. Given the identification of MDR bacteria on an antibiotic-free farm, it is important to understand the dynamics of gene exchange between naïve pathogens and non-pathogenic MDR reservoir species in the absence of selective antibiotic pressure. Comparison of our drug resistance profiles to the previous study indicates a potential exchange of resistances across bacterial barriers. Most notably, tetracycline resistance was found in all the previous genera tested, including *Campylobacter*, *Salmonella*, *Listeria*, and *Escherichia* and in both of our turkey-derived *A. lwoffii* isolates. Resistance to tetracycline is most commonly conferred by the activity of a drug efflux pump, which are typically found on mobile elements (Espinal et al., 2011). It will be interesting to sequence the genomes of the *A. lwoffii* and *A. johnsonii* isolates to compare the resistance profiles determined in this study with the genetic composition and extra chromosomal elements that these strains may possess. Additionally, the resistance to β-lactam derivatives was observed in the previous study, while resistance to cephalosporins, which was widespread in our study, was not (Rothrock et al., 2016). These resistance phenotypes can be effected by the activity of AmpC, a metallo-β-lactamase (MBL), or an oxacillinase, all of which have been reported to be associated with mobile genetic elements (Pfeifer et al., 2010; Evans and Amyes, 2014). We previously sequenced strain *A. radioresistens* LH6 described in this study. LH6 lacks plasmids, but encodes several putative MBLs, which can account for its resistance profile (Crippen et al., 2018). Interestingly, LH6 also encodes *bla*_OXA–2__3_ with 100% amino acid homology to the *A. baumannii* ASM74664v1 homolog, but no broad carbapenem, cephalosporin or β-lactamase resistance phenotype was observed (Table 1 and Supplementary Figure S4), which is normally associated with the presence of this gene (Pfeifer et al., 2010). This could be due to the lack of IMP-1, OXA-58, and IS*Acra1* in the LH6 genome, any of which are required for the trademark carbapenemase activity associated with the OXA-23 carbapenemase (Poirel et al., 2008; Higgins et al., 2013). Intermediate levels of nitrofurantoin resistance were found in *Listeria* spp. isolated in the Rothrock et al. (2016) study, while 9/10 of our isolates were resistant to nitrofurantoin, explained by the intrinsic nitrofurantoin resistance inherent to *Acinetobacter* spp. (Giske, 2015). This could be mediated by point mutations in *nsfA* or *nsfB*, which metabolize nitrofurantoin into the reactive intermediates that interfere with ribosomal subunits, or by *oxqAB* in some *Acinetobacter* spp., but the LH6 sequenced strain does not possess these efflux genes (Sekyere, 2018; Gardiner et al., 2019). Previously, a high incidence of fluoroquinolone resistance was also found among the *Listeria* spp., while aminoglycoside resistance was found in *E. coli* and *Salmonella* spp. (Rothrock et al., 2016). All of our isolates were susceptible to quinolone and aminoglycoside derivatives, indicating a barrier to genetic mobility that was potentially not present for tetracycline or β-lactam resistance elements. To further explore this phenomenon, we used a *gfp*-labeled resistance marker to demonstrate that plasmid DNA can be released through both contact-dependent killing by *A. baumannii* and by bacteriophage-mediated lysis.

In our study, we performed bacterial competition assays with two strains of *A. baumannii* and tested CDI and T6SS deletion mutants. Co-incubation of the isolated strains with *A. baumannii* strain 19606 and its isogenic *cdi1* and *cdi2* mutants resulted in extensive killing of *A. lwoffii*, and less with *A. radioresistens* and *A. johnsonii*. We observed that deletion of *cdi1* (Harding et al., 2017) did not increase survival of *A. johnsonii* and *A. radioresistens*, but did show less killing of *A. lwoffii*. In this study, *cdi2* mutation did not increase survival for any of the isolated strains. These results indicate that CDI systems have a minimal impact on *Acinetobacter* interspecies competition, except in the case of CDI1 and *A. lwoffii* strain D16. When using the *A. baumannii* 17978 strain and its isogenic *hcp* mutant, we observed minimal killing of *A. johnsonii*, while *A. radioresistens* and *A. lwoffii* were extensively killed and the T6SS could only account for part of this reduction. This killing could be due to an undescribed CDI system that does not resemble known CDI systems in that strain or other combat mechanisms expressed by the 17978 strain (Le et al., 2020). These recent findings are particularly relevant to consider in environments that are under constant selective pressure, such as the poultry gut (Singer and Hofacre, 2006). Rapid expansion of MDR has also been described in low-resource urban areas and rural farming areas where waste management is underdeveloped (Pehrsson et al., 2016). These observations make it important to understand the transfer of resistance determinants, prompting our subsequent experiments tracking an exogenously added resistance marker. We confirmed that release of the KmR gene was proportional to the amount of cell killing observed, consistent with observing the greatest reduction in *A. lwoffii* growth concomitant with the highest levels of KmR gene detection/release. Therefore, bacterial prey susceptibility to attack will likely impact the availability of new MDR genes for *A. baumannii* to acquire. Due to these combinations of selective pressures and diversity of bacterial inhabitants, reservoir species accumulate MDR genes that can be released by related pathogens such as *A. baumannii*, which has also been isolated from livestock in earlier studies (Fernando et al., 2016), and then incorporated into their own genomes.

Bacteriophages have also been described as vehicles for MDR spread through the transduction of genes or mobile elements that contain MDR genes (Brown-Jaque et al., 2015). If we consider the quantity of DNA that can be released when approximately half of the earth’s bacterial population is killed by bacteriophages every 2 days (Deresinski, 2009), then the exchange of MDR genes across species through phage release of DNA not only becomes likely, but inevitable. Evidence for this gene exchange was first demonstrated in the case of the *E. coli* “superspreader” bacteriophages (Keen et al., 2017). In addition to the findings of Keen et al. (2017), we have demonstrated the ability of the novel *A. radioresistens* bacteriophages CAP1 and CAP3 to release an intact KmR gene that could then potentially be incorporated by certain naturally competent bacteria that share the same environment, including *A. baumannii.* We further demonstrated this phenomenon by transforming the recovered DNA from phage-lysed cells into chemically competent *E. coli.* The probability that these two phage isolates, from different taxonomic families, are unique in their ability to release intact MDR genes is highly unlikely. Thus, great consideration must be given to all lytic phages when designing phage therapy treatments, because of the potential accelerated risk for horizontal gene transfer. These findings provide evidence for alternate methods for rapid MDR gene spread in bacterial species, especially in food animals. Additionally, these types of studies should be replicated for phage-derived elements such as endolysins, which are also being considered as potential therapeutics along with intact phages (Fischetti et al., 2006).

Because phage transduction can also be a mechanism for AMR determinant spread, we investigated the genomes of the isolated phages and their propagating strain, *A. radioresistens* LH6. Interestingly, 5/7 of the isolated phages were segmented RNA phages that are known to undergo recombination at high rates (Simon-Loriere and Holmes, 2011). In addition, genomic sequencing of *A. radioresistens* LH6 (Crippen et al., 2018) identified two possible chromosomal prophages, including one Mu-like, that may also be involved in AMR gene transduction (Braid et al., 2004). One of these prophages was indeed isolated after induction with mitomycin C and is being described in a separate manuscript (Crippen et al., in preparation). The presence of this active prophage and the regularity of prophages within the genus *Acinetobacter* indicates that prophages are also likely to participate in the dissemination of resistance determinants (Touchon et al., 2014; Costa et al., 2018).

Microbial antibiotic resistance is an ancient defense mechanism that is developing into a major crisis for healthcare providers due our great dependence on antibiotics for most medical procedures. The prevalence and mechanisms associated with antibiotic resistance are better characterized, but the mechanisms of MDR gene transfer in the environment are less understood. It is important to continue studying antibiotic resistance gene transfer, so that innovative solutions to the current antibiotic resistance crisis can be found (Bush et al., 2011; Culp et al., 2020).

## Data Availability Statement

All datasets generated for this study are included in the article/Supplementary Material.

## Author Contributions

CC performed all the experiments, with the exception of the strain identification and resistance profiles, and drafted the manuscript. MR provided access to the farm and the fecal samples that the bacterial strains and bacteriophages were isolated from. SS facilitated the strain identification and determination of resistance profiles. CS coordinated all the experiments. All authors edited the manuscript.

## Conflict of Interest

The authors declare that the research was conducted in the absence of any commercial or financial relationships that could be construed as a potential conflict of interest.

## Footnotes

1 http://www.eucast.org/clinical_breakpoints/
