# Supplementary material for: Multidrug Resistant Acinetobacter Isolates Release Resistance Determinants Through Contact-Dependent Killing and Bacteriophage Lysis
Source: Front Microbiol. 2020 Aug 14;11:1918. doi: 10.3389/fmicb.2020.01918 (PMC7456956; doi:10.3389/fmicb.2020.01918)
Supplement: Supplementary file 1 [file Data_Sheet_1.pdf]

Multidrug resistant *Acinetobacter* isolates release resistance determinants through contact-dependent killing and bacteriophage lysis

Clay S. Crippen[1], Michael J. Rothrock Jr.[2], Susan Sanchez[3] and Christine M. Szymanski[1]*

[1]Department of Microbiology and Complex Carbohydrate Research Center, University of Georgia, Athens, GA, USA

[2]Agricultural Research Service, United States Department of Agriculture, US National Poultry Research Center, Athens, GA, USA

[3]Department of Infectious Diseases and Athens Veterinary Diagnostic Lab, University of Georgia, Athens, GA, USA

Contained:

Supplementary Sequencing Data

16S rRNA sequences

>A17-37276 D16 @6/12/2017
GGTAGCGTCCTCCTTGCGGTTAGACTACCTACTTCTGGTGCAACAAACTCCCATGGTGTGA
CGGGCGGTGTGTACAAGGCCCGGGAACGTATTCACCGCGGCATTCTGATCCGCGATTACT
AGCGATTCCGACTTCATGGAGTCGAGTTGCAGACTCCAATCCGGACTACGATCGGCTTTTT
GAGATTAGCATCCTCTCGCGAGGTAGCAACCCTTTGTACCGACCATTGTAGCACGTGTGTA
GCCCTGGTCGTAAGGGCCATGATGACTTGACGTCGTCCCCGCCTTCCTCCAGTTTGTCAC
TGGCAGTATCCTTAAAGTTCCCGGCTTAACCCGCTGGCAAATAAGGAAAAGGGTTGCGCTC
GTTGCGGGACTTAACCCAACATCTCACGACACGAGCTGACGACAGCCATGCAGCACCTGT
ATGTAAGTTCCCGAAGGCACCAATCCATCTCTGGAAAGTTCTTACTATGTCAAGACCAGGT
AAGGTTCTTCGCGTTGCATCGAATTAAACCACATGCTCCACCGCTTGTGCGGGCCCCCGT
CAATTCATTTGAGTTTTAGTCTTGCGACCGTACTCCCCAGGCGGTCTACTTATCGCGTTAG
CTGCGCCACTAAAGCCTCAAAGGCCCCAACGGCTAGTAGACATCGTTTACGGCATGGACT
ACCAGGGTATCTAATCCTGTTTGCTCCCCATGCTTTCGCACCTCAGTGTCAGTATTAGGCC
AGATGGCTGCCTTCGCCATCGGTATTCCTCCAGATCTCTACGCATTTCACCGCTACACCTG
GAATTCTACCATCCTCTCCCATACTCTAGCCAACCAGTATCGAATGCAATTCCCAAGTTAAG
CTCGGGGATTTCACATTTGACTTAATTGGCCACCTACGCGCGCTTTACGCCCAGTAAATCC
GATTAACGCTTGCACCCTCTGTATTACCGCGGCTGCTGGCACAGAGTTAGCCGGTGCTTAT
TCTGCGAGTAACGTCCACTATCCAAGAGTATTAATCTCGGTAGCCTCCTCCTCGCTTAAAG
TGCTTTACAACCAAAAGGCCTTCTTCACACACGCGGCATGGCTGGATCAGGGTTCCCCC
ATTGTCCAATATTCCCCACTGCTGCCTCCCGTAGGAGTCTGGGCCGTGTCTCAGTCCCAGT
GTGGCGGATCATCCTCTCAGACCCGCTACAGATCGTCGCCTTGGTAGGCCTTTACCCCAC
CAACTAGCTAATCCGACTTAGGCTCATCTATTAGCGCAAGGCCCGAAGGTCCCCTGCTTTC
TCCCGTAGGACGTATGCGGTATTAGCATCCCTTTCGAGATGTTGTCCCCCACTAATAGGCA

GATTCCTAAGCATTACTCACCCGTCCGCCGCTAGGTTAGGTAGCAAGCTACCTT

>A17-37276 LH20-1 @6/12/2017
GCGTCCTCCTTGCGGTNNAGACTNNCCTACTTCTGGTGCAACAAACTCCCATGGTGTGAC
GGGCGGTGTGTACAAGGCCCGGGAACGTATTCACCGCGGCATTCTGATCCGCGATTACTA
GCGATTCCGACTTCATGGAGTCGAGTTGCAGACTCCAATCCGGACTACGATCGGCTTTTTG
AGATTAGCATCCTCTCGCGAGGTAGCAACCCTTTGTACCGACCATTGTAGCACGTGTGTAG
CCCTGGTCGTAAGGGCCATGATGACTTGACGTCGTCCCCGCCTTCCTCCAGTTTGTCACT
GGCAGTATCCTTAAAGTTCCCGGCTTAACCCGCTGGCAAATAAGGAAAAGGGTTGCGCTC
GTTGCGGGACTTAACCCAACATCTCACGACACGAGCTGACGACAGCCATGCAGCACCTGT
ATGTAAGTTCCCGAAGGCACCAATCCATCTCTGGAAAGTTCTTACTATGTCAAGACCAGGT
AAGGTTCTTCGCGTTGCATCGAATTAAACCACATGCTCCACCGCTTGTGCGGGCCCCCGT
CAATTCATTTGAGTTTTAGTCTTGCGACCGTACTCCCCAGGCGGTCTACTTATCGCGTTAG
CTGCGCCACTAAAGCCTCAAAGGCCCCAACGGCTAGTAGACATCGTTTACGGCATGGACT
ACCAGGGTATCTAATCCTGTTTGCTCCCCATGCTTTCGCACCTCAGTGTCAGTATTAGGCC
AGATGGCTGCCTTTCGCCATCGGTATTCCTCCAGATCTCTACGCATTTCACCGCTACACCT
GGANTTCTACCNNNNTCTCCCATACTCTAGCCAACCAGTATCGAATGCAATTCCCAAGTTA
AGCTCGGGGATTTCACATTTGACTTAATTGGCCACCTACGCGCGCTTTACGCCCAGTAAAT
CCGATTAACGCTTGCACCCTCTGTATTACCGCGGCTGCTGGCACAGAGTTAGCCGGTGCT
TATTCTGCGAGTAACGTCCACTATCCAAGAGTATTAATCTCGGTAGCCTCCTCCTCGCTTAA
AGTGCTTTACAACCAAAAGGCCTTCTTCACACACGCGGCATGGCTGGATCAGGGTTCCCC
CCATTGTCCAATATTCCCCACTGCTGCCTCCCGTAGGAGTCTGGGCCGTGTCTCAGTCCCA
GTGTGGCGGATCATCCTCTCAGACCCGCTACAGATCGTCGCCTTGGTAGGCCTTTACCCC
ACCAACTAGCTAATCCGACTTAGGCTCATCTATTAGCGCAAGGCCCGAAGGTCCCCTGCTT
TCTCCCGTAGGACGTATGCGGTATTAGCATCCCTTTCGAGATGTTGTCCCCC

>A17-37276 LH20-2 @6/12/2017
AGCGTCCTCCTTGCGGTTAGACTACCTACTTCTGGTGCAACAAACTCCCATGGTGTGACGG
GCGGTGTGTACAAGGCCCGGGAACGTATTCACCGCGGCATTCTGATCCGCGATTACTAGC
GATTCCGACTTCATGGAGTCGAGTTGCAGACTCCAATCCGGACTACGATCGGCTTTTTGAG
ATTAGCATCCTCTCGCGAGGTAGCAACCCTTTGTACCGACCATTGTAGCACGTGTGTAGCC
CTGGTCGTAAGGGCCATGATGACTTGACGTCGTCCCCGCCTTCCTCCAGTTTGTCACTGG
CAGTATCCTTAAAGTTCCCGGCTTAACCCGCTGGCAAATAAGGAAAAGGGTTGCGCTCGTT
GCGGGACTTAACCCAACATCTCACGACACGAGCTGACGACAGCCATGCAGCACCTGTATG
TAAGTTCCCGAAGGCACCAATCCATCTCTGGAAAGTTCTTACTATGTCAAGACCAGGTAAG
GTTCTTCGCGTTGCATCGAATTAAACCACATGCTCCACCGCTTGTGCGGGCCCCCGTCAAT
TCATTTGAGTTTTAGTCTTGCGACCGTACTCCCCAGGCGGTCTACTTATCGCGTTAGCTGC
GCCACTAAAGCCTCAAAGGCCCCAACGGCTAGTAGACATCGTTTACGGCATGGACTACCA
GGGTATCTAATCCTGTTTGCTCCCCATGCTTTCGCACCTCAGTGTCAGTATTAGGCCAGAT
GGCTGCCTTCGCCATCGGTATTCCTCCAGATCTCTACGCATTTCACCGCTACACCTGGAAT
TCTACCATCCTCTCCCATACTCTAGCCAACCAGTATCGAATGCAATTCCCAAGTTAAGCTCG
GGGATTTCACATTTGACTTAATTGGCCACCTACGCGCGCTTTACGCCCAGTAAATCCGATT
AACGCTTGCACCCTCTGTATTACCGCGGCTGCTGGCACAGAGTTAGCCGGTGCTTATTCTG
CGAGTAACGTCCACTATCCAAGAGTATTAATCTCGGTAGCCTCCTCCTCGCTTAAAGTGCT
TTACAACCAAAAGGCCTTCTTCACACACGCGGCATGGCTGGATCAGGGTTCCCCCATTGT
CCAATATTCCCCACTGCTGCCTCCCGTAGGAGTCTGGGCCGTGTCTCAGTCCCAGTGTGG
CGGATCATCCTCTCAGACCCGCTACAGATCGTCGCCTTGGTAGGCCTTTACCCCACCAACT
AGCTAATCCGACTTAGGCTCATCTATTAGCGCAAGGCCCGAAGGTCCCCTGCTTTCTCCCG

TAGGACGTATGCGGTATTAGCATCCCTTTCGAGATGTTGTCCCCCACTAATAGGCAGATTC
CTAAGCAT

>A17-37276 T10-1 @6/8/17
GNGGTAGCGTCCTCCTTGCGGTTAGACTACCTACTTCTGGTGCAACAAACTCCCATGGTGT
GACGGGCGGTGTGTACAAGGCCCGGGAACGTATTCACCGCGGCATTCTGATCCGCGATTA
CTAGCGATTCCGACTTCATGGAGTCGAGTTGCAGACTCCAATCCGGACTACGATCGGCTTT
TTGAGATTAGCATCCTATCGCTAGGTAGCAACCCTTTGTACCGACCATTGTAGCACGTGTG
TAGCCCTGGTCGTAAGGGCCATGATGACTTGACGTCGTCCCCGCCTTCCTCCAGTTTGTCA
CTGGCAGTATCCTTAAAGTTCCCGGCTTAACCCGCTGGCAAATAAGGAAAAGGGTTGCGCT
CGTTGCGGGACTTAACCCAACATCTCACGACACGAGCTGACGACAGCCATGCAGCACCTG
TATGTAAGTTCCCGAAGGCACCAATCCATCTCTGGAAAGTTCTTACTATGTCAAGACCAGG
TAAGGTTCTTCGCGTTGCATCGAATTAAACCACATGCTCCACCGCTTGTGCGGGCCCCCGT
CAATTCATTTGAGTTTTAGTCTTGCGACCGTACTCCCCAGGCGGTCTACTTATCGCGTTAG
CTGCGCCACTAAAGCCTCAAAGGCCCCAACGGCTAGTAGACATCGTTTACGGCATGGACT
ACCAGGGTATCTAATCCTGTTTGCTCCCCATGCTTTCGCACCTCAGTGTCAGTATTAGGCC
AGATGGCTGCCTTCGCCATCGGTATTCCTCCAGATCTCTACGCATTTCACCGCTACACCTG
GAATTCTACCATCCTCTCCCATACTCTAGCCAACCAGTATCGAATGCAATTCCCAAGTTAAG
CTCGGGGATTTCACATTTGACTTAATTGGCCACCTACGCGCGCTTTACGCCCAGTAAATCC
GATTAACGCTTGCACCCTCTGTATTACCGCGGCTGCTGGCACAGAGTTAGCCGGTGCTTAT
TCTGCGAGTAACGTCCAAGCATCTTGGGTATTAACCAAGAGCTCCTCCTCCTCGCTTAAAG
TGCTTTACAACCAAAAGGCCTTCTTCACACACGCGGCATGGCTGGATCAGGGTTCCCCCC
ATTGTCCAATATTCCCCACTGCTGCCTCCCGTAGGAGTCTGGGCCGTGTCTCAGTCCCAGT
GTGGCGGATCATCCTCTCAGACCCGCTACAGATCGTCGCCTTGGTAGGCCTTTACCCCAC
CAACTAGCTAATCCGACTTAGGCTCATCTATTAGCGCAAGGTCCGAAGATCCCCTGCTTTC
TCCCGTAGGACGTATGCGGTATTAGCATCCCTTTCGAGATGTTGTCCCCCACTAATAGGCA
GATTCCTAAGCATTACTCACCCGTCCGCCG

>A17-37276 T10-2 @6/8/2017
GNGNACAAGGCCCGGGAACGTATTCACCGCGGCATTCTGATCCGCGATTACTAGCGATTC
CGACTTCATGGAGTCGAGTTGCAGACTCCAATCCGGACTACGATCGGCTTTTTGAGATTAG
CATCCTATCGCTAGGTAGCAACCCTTTGTACCGACCATTGTAGCACGTGTGTAGCCCTGGT
CGTAAGGGCCATGATGACTTGACGTCGTCCCCGCCTTCCTCCAGTTTGTCACTGGCAGTAT
CCTTAAAGTTCCCGGCTTAACCCGCTGGCAAATAAGGAAAAGGGTTGCGCTCGTTGCGGG
ACTTAACCCAACATCTCACGACACGAGCTGACGACAGCCATGCAGCACCTGTATGTAAGTT
CCCGAAGGCACCAATCCATCTCTGGAAAGTTCTTACTATGTCAAGACCAGGTAAGGNTCTT
CGCGTTGCATCGAATTAAACCACATGCTCCACCGCTTGTGCGGGCCCCCGTCAATTCATTT
GAGTTTTAGTCTTGCGACCGTACTCCCCAGGCGGTCTACTTATCGCGTTAGCTGCGCCACT
AAAGCCTCAAAGGCCCCAACGGCTAGTAGACATCGTTTACGGCATGGACTACCAGGGTAT
CTAATCCTGTTTGCTCCCCATGCTTTCGCACCTCAGTGTCAGTATTAGGCCAGATGGCTGC
CTTCGCCATCGGTATTCCTCCAGATCTCTACGCATTTCACCGCTACACCTGGNAATTCTAC
CATCCTCTCCCATACTCTAGCCAACCAGTATCGAATGCAATTCCCAAGTTAAGCTCGGGGA
TTTCACATTTGACTTAATTGGCCACCTACGCGCGCTTTACGCCCAGTAAATCCGATTAACGC
TTGCACCCTCTGTATTACCGCGGCTGCTGGCACAGAGTTAGCCGGTGCTTATTCTGCGAGT
AACGTCCAAGCATCTTGGGTATTAACCAAGAGCTCCTCCTCCTCGCTTAAAGTGCTTTACAA
CCAAAAGGCCTTCTTCACACACGCGGCATGGCTGGATCAGGGTTCCCCCCATTGTCCAAT
ATTCCCCACTGCTGCCTCCCGTAGGAGTCTGGGCCGTGTCTCAGTCCCAGTGTGGCGGAT
CATCCTCTCAGACCCGCTACAGATCGTCGCCTTGGTAGGCCTTTACCCCACCAACTAGCTA

ATCCGACTTAGGCTCATCTATTAGCGCAAGGTCCGAAGATCCCCTGCTTTCTCCCGTAGGA
CGTATGCGGTATTAGCATCCCTTTCGAGATGTTGTCCCCCACTAATAGGCAGATTCCTAAG
CATTACTCACCCGTCCGCCGC

>A17-37276 T8@6/8/2017
GNAGCGTCCTCCTTGCGGTTAGACTACCTACTTCTGGTGCAACAAACTCCCATGGTGTGAC
GGGCGGTGTGTACAAGGCCCGGGAACGTATTCACCGCGGCATTCTGATCCGCGATTACTA
GCGATTCCGACTTCATGGAGTCGAGTTGCAGACTCCAATCCGGACTACGATCGGCTTTTTG
AGATTAGCATCCTATCGCTAGGTAGCAACCCTTTGTACCGACCATTGTAGCACGTGTGTAG
CCCTGGTCGTAAGGGCCATGATGACTTGACGTCGTCCCCGCCTTCCTCCAGTTTGTCACT
GGCAGTATCCTTAAAGTTCCCGGCTTAACCCGCTGGCAAATAAGGAAAAGGGTTGCGCTC
GTTGCGGGACTTAACCCAACATCTCACGACACGAGCTGACGACAGCCATGCAGCACCTGT
ATGTAAGTTCCCGAAGGCACCAATCCATCTCTGGAAAGTTCTTACTATGTCAAGACCAGGT
AAGGTTCTTCGCGTTGCATCGAATTAAACCACATGCTCCACCGCTTGTGCGGGCCCCCGT
CAATTCATTTGAGTTTTAGTCTTGCGACCGTACTCCCCAGGCGGTCTACTTATCGCGTTAG
CTGCGCCACTAAAGCCTCAAAGGCCCCAACGGCTAGTAGACATCGTTTACGGCATGGACT
ACCAGGGTATCTAATCCTGTTTGCTCCCCATGCTTTCGCACCTCAGTGTCAGTATTAGGCC
AGATGGCTGCCTTCGCCATCGGTATTCCTCCAGATCTCTACGCATTTCACCGCTACACCTG
GAATTCTACCATCCTCTCCCATACTCTAGCCAACCAGTATCGAATGCAATTCCCAAGTTAAG
CTCGGGGATTTCACATTTGACTTAATTGGCCACCTACGCGCGCTTTACGCCCAGTAAATCC
GATTAACGCTTGCACCCTCTGTATTACCGCGGCTGCTGGCACAGAGTTAGCCGGTGCTTAT
TCTGCGAGTAACGTCCACTATCCCTAGGTATTAACTAGAGTAGCCTCCTCCTCGCTTAAAG
TGCTTTACAACCAAAAGGCCTTCTTCACACACGCGGCATGGCTGGATCAGGGTTCCCCCC
ATTGTCCAATATTCCCCACTGCTGCCTCCCGTAGGAGTCTGGGCCGTGTCTCAGTCCCAGT
GTGGCGGATCATCCTCTCAGACCCGCTACAGATCGTCGCCTTGGTAGGCCTTTACCCCAC
CAACTAGCTAATCCGACTTAGGCTCATCTATTAGCGCAAGGTCCGAAGATCCCCTGCTTTC
TCCCGTAGGACGTATGCGGTATTAGCATCCCTTTCGAGATGTTGTCCCCCACTAATAGGCA
GATTCCTAAGCATTACTCACCCGTCCGCCGCTAGG

>A17-37276 D16-1492R
GGTAGCGTCCTCCTTGCGGTTAGACTACCTACTTCTGGTGCAACAAACTCCCATGGTGTGA
CGGGCGGTGTGTACAAGGCCCGGGAACGTATTCACCGCGGCATTCTGATCCGCGATTACT
AGCGATTCCGACTTCATGGAGTCGAGTTGCAGACTCCAATCCGGACTACGATCGGCTTTTT
GAGATTAGCATCCTCTCGCGAGGTAGCAACCCTTTGTACCGACCATTGTAGCACGTGTGTA
GCCCTGGTCGTAAGGGCCATGATGACTTGACGTCGTCCCCGCCTTCCTCCAGTTTGTCAC
TGGCAGTATCCTTAAAGTTCCCGGCTTAACCCGCTGGCAAATAAGGAAAAGGGTTGCGCTC
GTTGCGGGACTTAACCCAACATCTCACGACACGAGCTGACGACAGCCATGCAGCACCTGT
ATGTAAGTTCCCGAAGGCACCAATCCATCTCTGGAAAGTTCTTACTATGTCAAGACCAGGT
AAGGTTCTTCGCGTTGCATCGAATTAAACCACATGCTCCACCGCTTGTGCGGGCCCCCGT
CAATTCATTTGAGTTTTAGTCTTGCGACCGTACTCCCCAGGCGGTCTACTTATCGCGTTAG
CTGCGCCACTAAAGCCTCAAAGGCCCCAACGGCTAGTAGACATCGTTTACGGCATGGACT
ACCAGGGTATCTAATCCTGTTTGCTCCCCATGCTTTCGCACCTCANTGTCAGTATTAGGCC
AGATGGCTGCCTTCGCCATCGGTATTCCTCCAGATCTCT

>A17-37276_D16-8F
TCGTTTACGGCATGGACTACCAGGGTATCTAATCCTGTTTGCTCCCCATGCTTTCGCACCT
CAGTGTCAGTATTAGGCCAGATGGCTGCNTTCGCCATCGGTATTCCTCCAGATCTCTACGC
ATTTCACCGCTACACCTGGAATTCTACCATCCTCTCCCATACTCTAGCCAACCAGTATCGAA
TGCAATTCCCAAGTTAAGCTCGGGGATTTCACATTTGACTTAATTGGCCACCTACGCGCGC

TTTACGCCCAGTAAATCCGATTAACGCTTGCACCCTCTGTATTACCGCGGCTGCTGGCACA
GAGTTAGCCGGTGCTTATTCTGCGAGTAACGTCCACTATCCAAGAGTATTAATCTCGGTAG
CCTCCTCCTCGCTTAAAGTGCTTTACAACCAAAAGGCCTTCTTCACACACGCGGCATGGCT
GGATCAGGGTTCCCCCCATTGTCCAATATTCCCCACTGCTGCCTCCCGTAGGAGTCTGGG
CCGTGTCTCAGTCCCAGTGTGGCGGATCATCCTCTCAGACCCGCTACAGATCGTCGCCTT
GGTAGGCCTTTACCCCACCAACTAGCTAATCCGACTTAGGCTCATCTATTAGCGCAAGGCC
CGAAGGTCCCCTGCTTTCTCCCGTAGGACGTATGCGGTATTAGCATCCCTTTCGAGATGTT
GTCCCCCACTAATAGGCAGATTCCTAAGCATTACTCACCCGTCCGCCGCTAGGTTAGGTAG
CAAGCTACCTT

>A17-37276_LH20-1-1492R
GCGTCCTCCTTGCGGTNNAGACTNNCCTACTTCTGGTGCAACAAACTCCCATGGTGTGAC
GGGCGGTGTGTACAAGGCCCGGGAACGTATTCACCGCGGCATTCTGATCCGCGATTACTA
GCGATTCCGACTTCATGGAGTCGAGTTGCAGACTCCAATCCGGACTACGATCGGCTTTTTG
AGATTAGCATCCTCTCGCGAGGTAGCAACCCTTTGTACCGACCATTGTAGCACGTGTGTAG
CCCTGGTCGTAAGGGCCATGATGACTTGACGTCGTCCCCGCCTTCCTCCAGTTTGTCACT
GGCAGTATCCTTAAAGTTCCCGGCTTAACCCGCTGGCAAATAAGGAAAAGGGTTGCGCTC
GTTGCGGGACTTAACCCAACATCTCACGACACGAGCTGACGACAGCCATGCAGCACCTGT
ATGTAAGTTCCCGAAGGCACCAATCCATCTCTGGAAAGTTCTTACTATGTCAAGACCAGGT
AAGGTTCTTCGCGTTGCATCGAATTAAACCACATGCTCCACCGCTTGTGCGGGCCCCCGT
CAATTCATTTGAGTTTTAGTCTTGCGACCGTACTCCCCAGGCGGTCTACTTATCGCGTTAG
CTGCGCCACTAAAGCCTCAAAGGCCCCAACGGCTAGTAGACATCGTTTACGGCATGGACT
ACCAGGGTATCTAATCCTGTTTGCTCCCCATGCTTTCGCACCTCANTGTCAGTATTAGGCC
AGATGGCTGCCTTCGCCATCGGTATTCCTCCAGATCTCTACGCNTTTCACCGCTACACCTG
G

>A17-37276-LH20-1-8F
TCAATTCATNGAGNTTANTNTNCGACCGTACNCCCCNGGCGGTCTACTNATCGCNNNGNT
GCGCCACTAAAGCCTCAAAGNCCCCAACGGCTAGTAGACATCGTTTACGGCATGGACTAC
CAGGGTATCTANTCCTGTTTGCTCCCCATGCTTTCGCACCTCAGTGTCAGTATTAGNCCAG
ATGGCTNCCTTTCNCCATCGGTATTCCTCCANATCTCTACGCATTTCACCGCTACACCTGG
ANTTCTACCNNNNTCTCCCATACTCTAGCCAACCAGTATCGAATGCAATTCCCAAGTTAAGC
TCGGGGATTTCACATTTGACTTAATTGGCCACCTACGCGCGCTTTACGCCCAGTAAATCCG
ATTAACGCTTGCACCCTCTGTATTACCGCGGCTGCTGGCACAGAGTTAGCCGGTGCTTATT
CTGCGAGTAACGTCCACTATCCAAGAGTATTAATCTCGGTAGCCTCCTCCTCGCTTAAAGT
GCTTTACAACCAAAAGGCCTTCTTCACACACGCGGCATGGCTGGATCAGGGTTCCCCCCA
TTGTCCAATATTCCCCACTGCTGCCTCCCGTAGGAGTCTGGGCCGTGTCTCAGTCCCAGT
GTGGCGGATCATCCTCTCAGACCCGCTACAGATCGTCGCCTTGGTAGGCCTTTACCCCAC
CAACTAGCTAATCCGACTTAGGCTCATCTATTAGCGCAAGGCCCGAAGGTCCCCTGCTTTC
TCCCGTAGGACGTATGCGGTATTAGCATCCCTTTCGAGATGTTGTCCCCC

>A17-37276-LH20-2-1492R
AGCGTCCTCCTTGCGGTTAGACTACCTACTTCTGGTGCAACAAACTCCCATGGTGTGACGG
GCGGTGTGTACAAGGCCCGGGAACGTATTCACCGCGGCATTCTGATCCGCGATTACTAGC
GATTCCGACTTCATGGAGTCGAGTTGCAGACTCCAATCCGGACTACGATCGGCTTTTTGAG
ATTAGCATCCTCTCGCGAGGTAGCAACCCTTTGTACCGACCATTGTAGCACGTGTGTAGCC
CTGGTCGTAAGGGCCATGATGACTTGACGTCGTCCCCGCCTTCCTCCAGTTTGTCACTGG
CAGTATCCTTAAAGTTCCCGGCTTAACCCGCTGGCAAATAAGGAAAAGGGTTGCGCTCGTT
GCGGGACTTAACCCAACATCTCACGACACGAGCTGACGACAGCCATGCAGCACCTGTATG

TAAGTTCCCGAAGGCACCAATCCATCTCTGGAAAGTTCTTACTATGTCAAGACCAGGTAAG
GTTCTTCGCGTTGCATCGAATTAAACCACATGCTCCACCGCTTGTGCGGGCCCCCGTCAAT
TCATTTGAGTTTTAGTCTTGCGACCGTACTCCCCAGGCGGTCTACTTATCGCGTTAGCTGC
GCCACTAAAGCCTCAAAGGCCCCAACGGCTAGTAGACATCGTTTACGGCATGGACTACCA
GGGTATCTAATCCTGTTTGCTCCCCATGCTTTCGCACCTCANTGTCAGTATTAGGCCAGAT
GGCTGCCTTCGCCATCGGTATTCCTCCAGATCTCTACGCNNTTCACCGCTACACCTGGAAT
TCTACCATCCTCTCCCATACTCTAGCCAACCAGTATCGAA

>A17-37276-LH20-2-8F
TAGCTGCGCCACTAAAGCCTCAAAGGCCCCAACGGCTAGTAGACATCNNNACGGCATGGA
CTACCAGGGTATCTAATCCTGTTTGCTCCCCATGCTTTCGCACCTCAGTGTCAGTATTAGG
CCAGATGGCTNNNTNCGCCATCGGTATTCCTCCAGATCTCTACGCATTTCACCGCTACACC
TGGAATTCTACCATCCTCTCCCATACTCTAGCCAACCAGTATCGAATGCAATTCCCAAGTTA
AGCTCGGGGATTTCACATTTGACTTAATTGGCCACCTACGCGCGCTTTACGCCCAGTAAAT
CCGATTAACGCTTGCACCCTCTGTATTACCGCGGCTGCTGGCACAGAGTTAGCCGGTGCT
TATTCTGCGAGTAACGTCCACTATCCAAGAGTATTAATCTCGGTAGCCTCCTCCTCGCTTAA
AGTGCTTTACAACCAAAAGGCCTTCTTCACACACGCGGCATGGCTGGATCAGGGTTCCCC
CCATTGTCCAATATTCCCCACTGCTGCCTCCCGTAGGAGTCTGGGCCGTGTCTCAGTCCCA
GTGTGGCGGATCATCCTCTCAGACCCGCTACAGATCGTCGCCTTGGTAGGCCTTTACCCC
ACCAACTAGCTAATCCGACTTAGGCTCATCTATTAGCGCAAGGCCCGAAGGTCCCCTGCTT
TCTCCCGTAGGACGTATGCGGTATTAGCATCCCTTTCGAGATGTTGTCCCCCACTAATAGG
CAGATTCCTAAGCAT

>A17-37276-T10-1-1492R
GNGGTAGCGTCCTCCTTGCGGTTAGACTACCTACTTCTGGTGCAACAAACTCCCATGGTGT
GACGGGCGGTGTGTACAAGGCCCGGGAACGTATTCACCGCGGCATTCTGATCCGCGATTA
CTAGCGATTCCGACTTCATGGAGTCGAGTTGCAGACTCCAATCCGGACTACGATCGGCTTT
TTGAGATTAGCATCCTATCGCTAGGTAGCAACCCTTTGTACCGACCATTGTAGCACGTGTG
TAGCCCTGGTCGTAAGGGCCATGATGACTTGACGTCGTCCCCGCCTTCCTCCAGTTTGTCA
CTGGCAGTATCCTTAAAGTTCCCGGCTTAACCCGCTGGCAAATAAGGAAAAGGGTTGCGCT
CGTTGCGGGACTTAACCCAACATCTCACGACACGAGCTGACGACAGCCATGCAGCACCTG
TATGTAAGTTCCCGAAGGCACCAATCCATCTCTGGAAAGTTCTTACTATGTCAAGACCAGG
TAAGGTTCTTCGCGTTGCATCGAATTAAACCACATGCTCCACCGCTTGTGCGGGCCCCCGT
CAATTCATTTGAGTTTTAGTCTTGCGACCGTACTCCCCAGGCGGTCTACTTATCGCGTTAG
CTGCGCCACTAAAGCCTCAAAGGCCCCAACGGCTAGTAGACATCGTTTACGGCATGGACT
ACCAGGGTATCTAATCCTGTTTGCTCCCCATGCTTTCGCACCTCANTGTCAGTATTAGGCC
AGATGGCTGCCTTCGCCATCGGTATTCCTCCAGATCTCTA

>A17-37276-T10-1-8F
GNCCCCAACGGCTAGTAGACATCGTTTACGGCATGGACTACCAGGGTATCTAATCCTGTTT
GCTCCCCATGCTTTCGCACCTCAGTGTCAGTATTAGGCCAGATGGCTGCNTTCGCCATCG
GTATTCCTCCAGATCTCTACGCATTTCACCGCTACACCTGGAATTCTACCATCCTCTCCCAT
ACTCTAGCCAACCAGTATCGAATGCAATTCCCAAGTTAAGCTCGGGGATTTCACATTTGACT
TAATTGGCCACCTACGCGCGCTTTACGCCCAGTAAATCCGATTAACGCTTGCACCCTCTGT
ATTACCGCGGCTGCTGGCACAGAGTTAGCCGGTGCTTATTCTGCGAGTAACGTCCAAGCA
TCTTGGGTATTAACCAAGAGCTCCTCCTCCTCGCTTAAAGTGCTTTACAACCAAAAGGCCTT
CTTCACACACGCGGCATGGCTGGATCAGGGTTCCCCCATTGTCCAATATTCCCCACTGCT
GCCTCCCGTAGGAGTCTGGGCCGTGTCTCAGTCCCAGTGTGGCGGATCATCCTCTCAGAC
CCGCTACAGATCGTCGCCTTGGTAGGCCTTTACCCCACCAACTAGCTAATCCGACTTAGGC

TCATCTATTAGCGCAAGGTCCGAAGATCCCCTGCTTTCTCCCGTAGGACGTATGCGGTATT
AGCATCCCTTTCGAGATGTTGTCCCCCACTAATAGGCAGATTCCTAAGCATTACTCACCCG
TCCGCCG

>A17-37276-T10-2-1492R
GNGNACAAGGCCCGGGAACGTATTCACCGCGGCATTCTGATCCGCGATTACTAGCGATTC
CGACTTCATGGAGTCGAGTTGCAGACTCCAATCCGGACTACGATCGGCTTTTTGAGATTAG
CATCCTATCGCTAGGTAGCAACCCTTTGTACCGACCATTGTAGCACGTGTGTAGCCCTGGT
CGTAAGGGCCATGATGACTTGACGTCGTCCCCGCCTTCCTCCAGTTTGTCACTGGCAGTAT
CCTTAAAGTTCCCGGCTTAACCCGCTGGCAAATAAGGAAAAGGGTTGCGCTCGTTGCGGG
ACTTAACCCAACATCTCACGACACGAGCTGACGACAGCCATGCAGCACCTGTATGTAAGTT
CCCGAAGGCACCAATCCATCTCTGGAAAGTTCTTACTATGTCAAGACCAGGTAAGGNTCTT
CGCGTTGCATCGAATTAAACCACATGCTCCACCGCTTGTGCGGGCCCCCGTCAATTCATTT
GAGTTTTAGTCTTGCGACCGTACTCCCCAGGCGGTCTACTTATCGCGTTAGCTGCGCCACT
AAAGCCTCAAAGGCCCCAACGGCTAGTAGACATCGTTTACGGCATGGACTACCANGGTAT
CTAATCCTGTTTGCTCCCCATGCTTTCGCACCTCAGTGTCAGTATTAGGCCAGATGGCTGC
CTTCGCCATCGGTATTCCTCCANATCTCTACGCATTTCACCGCTACACCTGGNANTTCTAC

>A17-37276-T10-2-8F
TAGCTGCGCCACTAAAGCCTCAAAGGCCCCAACGGCTAGTAGACATCGTTTACGGCATGG
ACTACCAGGGTATCTANTCCTGTTTGCTCCCCATGCTTTCGCACCTCAGTGTCAGTATTAG
GCCAGATGGCTGCNTTCGCCATCGGTATTCCTCCAGATCTCTACGCATTTCACCGCTACAC
CTGGAATTCTACCATCCTCTCCCATACTCTAGCCAACCAGTATCGAATGCAATTCCCAAGTT
AAGCTCGGGGATTTCACATTTGACTTAATTGGCCACCTACGCGCGCTTTACGCCCAGTAAA
TCCGATTAACGCTTGCACCCTCTGTATTACCGCGGCTGCTGGCACAGAGTTAGCCGGTGC
TTATTCTGCGAGTAACGTCCAAGCATCTTGGGTATTAACCAAGAGCTCCTCCTCCTCGCTTA
AAGTGCTTTACAACCAAAAGGCCTTCTTCACACACGCGGCATGGCTGGATCAGGGTTCCC
CCCATTGTCCAATATTCCCCACTGCTGCCTCCCGTAGGAGTCTGGGCCGTGTCTCAGTCC
CAGTGTGGCGGATCATCCTCTCAGACCCGCTACAGATCGTCGCCTTGGTAGGCCTTTACC
CCACCAACTAGCTAATCCGACTTAGGCTCATCTATTAGCGCAAGGTCCGAAGATCCCCTGC
TTTCTCCCGTAGGACGTATGCGGTATTAGCATCCCTTTCGAGATGTTGTCCCCCACTAATA
GGCAGATTCCTAAGCATTACTCACCCGTCCGCCGC

>A17-37276-T8-1492R
GNAGCGTCCTCCTTGCGGTTAGACTACCTACTTCTGGTGCAACAAACTCCCATGGTGTGAC
GGGCGGTGTGTACAAGGCCCGGGAACGTATTCACCGCGGCATTCTGATCCGCGATTACTA
GCGATTCCGACTTCATGGAGTCGAGTTGCAGACTCCAATCCGGACTACGATCGGCTTTTTG
AGATTAGCATCCTATCGCTAGGTAGCAACCCTTTGTACCGACCATTGTAGCACGTGTGTAG
CCCTGGTCGTAAGGGCCATGATGACTTGACGTCGTCCCCGCCTTCCTCCAGTTTGTCACT
GGCAGTATCCTTAAAGTTCCCGGCTTAACCCGCTGGCAAATAAGGAAAAGGGTTGCGCTC
GTTGCGGGACTTAACCCAACATCTCACGACACGAGCTGACGACAGCCATGCAGCACCTGT
ATGTAAGTTCCCGAAGGCACCAATCCATCTCTGGAAAGTTCTTACTATGTCAAGACCAGGT
AAGGTTCTTCGCGTTGCATCGAATTAAACCACATGCTCCACCGCTTGTGCGGGCCCCCGT
CAATTCATTTGAGTTTTAGTCTTGCGACCGTACTCCCCAGGCGGTCTACTTATCGCGTTAG
CTGCGCCACTAAAGCCTCAAAGGCCCCAACGGCTAGTAGACATCGTTTACGGCATGGACT
ACCAGGGTATCTAATCCTGTTTGCTCCCCATGCTTTCGCACCTCANTGTCAGTATTAGGCC
AGATGGCTGCCTTCGCCATCGGTATTCCTCCAGATCTCTA

>A17-37276-T8-8F
CCCAGNCGGTCTACTTATCGCNTTAGCTGCGCCACTAAAGCCTCAAAGNCCCCAACGGCT
AGTAGACATCGTTTACGGCATGGACTACCAGGGTATCTAATCCTGTTTGCTCCCCATGCTT
TCGCACNTCAGTGTCAGTATTAGGCCAGATGGCTGCNTTCGCCATCGGTATTCCTCCAGAT
CTCTACGCATTTCACCGCTACACCTGGAATTCTACCATCCTCTCCCATACTCTAGCCAACCA
GTATCGAATGCAATTCCCAAGTTAAGCTCGGGGATTTCACATTTGACTTAATTGGCCACCTA
CGCGCGCTTTACGCCCAGTAAATCCGATTAACGCTTGCACCCTCTGTATTACCGCGGCTGC
TGGCACAGAGTTAGCCGGTGCTTATTCTGCGAGTAACGTCCACTATCCCTAGGTATTAACT
AGAGTAGCCTCCTCCTCGCTTAAAGTGCTTTACAACCAAAAGGCCTTCTTCACACACGCGG
CATGGCTGGATCAGGGTTCCCCCCATTGTCCAATATTCCCCACTGCTGCCTCCCGTAGGA
GTCTGGGCCGTGTCTCAGTCCCAGTGTGGCGGATCATCCTCTCAGACCCGCTACAGATCG
TCGCCTTGGTAGGCCTTTACCCCACCAACTAGCTAATCCGACTTAGGCTCATCTATTAGCG
CAAGGTCCGAAGATCCCCTGCTTTCTCCCGTAGGACGTATGCGGTATTAGCATCCCTTTCG
AGATGTTGTCCCCCACTAATAGGCAGATTCCTAAGCATTACTCACCCGTCCGCCGCTAGG

***rpoB* sequences**

>LH1
NCNACACCCNNNNNNNCCGTGACGACCCGCCATTTTATCACCCGGCTGNNGATACGACGT
TTAACTGCCAGATAAACCTTCACAACTTTCAATACGCCATGAGTCAGCTCATCACCCGTAGA
AAGCTTGCGTTTCTTCTCGGCAAACTTCTCGTCAATCTCGGTGCTTTTCTCTTTCAAGAACA
CCTGGATCTGGGTTAAACGTTCAGCAATCGCCTCATCGCTTGGCTGGATTTCGAGCAAATC
AATCAGTTCAAGGCCAGATAGCAATTCTTCAGACAGTTTCTCACCACGTTTGGTATTTCCAC
CGCCGTTAGACACCTGATCTTTCAGCAAACGCACAATACGTTCACGAGCCGCTTCTTCAAA
GATTTTATATTCTTCTTTNNNNNNNN

>LH2
TCNACACCCNNNNNNNNCGTGACGACCCGCCATCTTATCACCCGGTTGGATGCGACGTTT
AACAGCCAAGTAAACCTTAACAACTTTCAATACACCTGTTGTTAATTCATCACCTGTAGAAA
GTTTGCGTTTTTTCTCTGCAAATTTCTCATCAATTTCGAAGCTCTTCTCTTTCAAGAACACTT
GAATTTGAGTTAAACGCTCTGCAATAGCTTCATCAGTCGGTTGGATTTCAAGTAAATCAACC
AACTCTAAACCAGACAATACGTCTGCAGAAAGCTTATCGCCACGCTTAGTTGTACCACCAC
CGTTAGACTCTTGACCTGTCAACAAACGAACAATACGTTCACGTGCTGCTTCTTCGAAGATT
TTGTATTCTTCTTTNNNNNNNTTTNNGNNAA

>LH3
TCCAACNNCNNNNNNNCCGTGACGACCCGCCATTTTATCACCCGGCTGGATACGACGTTT
AACTGCCAGATAAACCTTCACAACTTTCAATACGCCATGAGTCAGCTCATCACCCGTAGAA
AGCTTGCGTTTCTTCTCGGCAAACTTCTCGTCAATCTCGGTGCTTTTCTCTTTCAAGAACAC
CTGGATCTGGGTTAAACGTTCAGCAATCGCCTCATCGCTTGGCTGGATTTCGAGCAAATCA
ATCAGTTCAAGGCCAGATAGCAATTCTTCAGACAGTTTCTCACCACGTTTGGTATTTCCACC
GCCGTTAGACACCTGATCTTTCAGCAAACGCACAATACGTTCACGAGCCGCTTCTTCAAAG
ATTTTATATTCTTCTTTCAAATNNTTTACNNTAA

>LH5
CCNNNCNGCGGGGGGGGGNNGGGNGNNGTNTNNCNCNNNNNCGGNGTNNNNAANNAAN
NANNNNNCNNNNNNNNNNNNANNNNNCNNNANANNNCNNANNNTNTACGGNNGNTGNGNG
NNNGANGGNGNNNGNNNNTNNGGGTTTTTTNNGGNNNAANANNGGNNNNNNNNNGGNG

ANAANNNNCNGGNNNTCCACACCCCNNNNNNCCGTGACGACCCGCCATTTTATCACCCGG
CTGGATACGACGTTTAACTGCCAGATAAACCTTCACAACTTTCAATACGCCATGAGTCAGCT
CATCACCCGTAGAAAGCTTGCGTTTCTTCTCGGCAAACTTCTCGTCAATCTCGGTGCTTTTC
TCTTTCAAGAACACCTGGATCTGGGTTAAACGTTCAGCAATCGCCTCATCGCTTGGCTGGA
TTTCGAGCAAATCAATCAGTTCAAGGCCAGATAGCAATTCTTCAGACAGTTTCTCACCACGT
TTGGTATTTCCACCGCCGTTAGACACCTGATCTTTCAGCAAACGCACAATACGTTCACGAG
CCGCTTCTTCAAAGATTTTATATTCTTCTTTCAAATN

>LH20_1
NNNCNACNCCNNNNNNNNCCGTGACGACCCGCCATCTTATCACCCGGCTGGATGCGACGTT
TCACAGCAAGATAAACTTTAACTACTTTCAATACGCCAGTCGTCAGTTCATCGCCTGTAGAA
AGTTTGCGTTTTTTCTCAGCAAATTTCTCATCAATTTCGAAGCTCTTCTCTTTCAAGAACACT
TGAATTTGAGTTAAACGTTCAGCAATTGCTTCGTCAACTGGTTGAATTTCAAGAAGATCAAC
AAGCTCTAAACCAGACAACACGTCTTCAGACAGTTTGTCGCCACGTTTAGTTGTACCGCCA
CCATTCGACTCTTGGCCGTTGAGTAGACGGATTACACGTTCACGTGCAGCTTCTTCGAAGA
TTTTGAATTCTTCTTTNNNNNNNNTTNNNNNAA

>LH20_2
TCNACACCNNNNNNNNNCCGTGACGACCCGCCATCTTATCACCCGGCTGNNGATGCGACGT
TTCACAGCAAGATAAACTTTAACTACTTTCAATACGCCAGTCGTCAGTTCATCGCCTGTAGA
AAGTTTGCGTTTTTTCTCAGCAAATTTCTCATCAATTTCGAAGCTCTTCTCTTTCAAGAACAC
TTGAATTTGAGTTAAACGTTCAGCAATTGCTTCGTCAACTGGTTGAATTTCAAGAAGATCAA
CAAGCTCTAAACCAGACAACACGTCTTCAGACAGTTTGTCGCCACGTTTAGTTGTACCGCC
ACCATTCGACTCTTGGCCGTTGAGTAGACGGATTACACGTTCACGTGCAGCTTCTTCGAAG
ATTTTGAATTCTTCTTTNNNNNNNNTTTACNNNAANN

>T8
TCNNCACCCNNNNNNNNCCGTGACGACCGGCCATCTTATCACCCGGCTGGATGCGACGTTT
TACCGCAAGATAAACTTTAACTACTTTCAATACGCCAGTGGTCAGCTCATCGCCTGTAGAAA
GTTTGCGTTTTTTCTCAGCAAATTTCTCGTCAATTTCGAAGCTCTTCTCTTTCAAGAACACTT
GAATTTGCGTTAGACGTTCAGCGATTGCTTCATCAACCGGTTGAATTTCAAGAAGATCAACA
AGCTCTAAACCAGACAACACGTCTTCAGACAGTTTGTCGCCACGTTTAGTGGTACCGCCAC
CATTCGACTCTTGACCGTTCAACAAACGGATTACACGTTCACGTGCAGCTTCTTCGAAGATT
TTGAATTCTTCTTTNNNNNNNNTTTACGNNNNNNNNNGGGTGTNGANNTGTNGANNNATAANA
TNGCCCNNNNNNCACNNNNACNNNNNTGTNNGANNA

>T10_1
TCNACACCNNNNNNNNNCCGTGACGACCGGCCATCTTATCACCCGGCTGGATGCGACGTTT
TACCGCAAGATAAACTTTAACTACTTTCAATACGCCAGTGGTCAGCTCATCGCCTGTAGAAA
GTTTGCGTTTTTTCTCAGCAAATTTCTCGTCAATTTCGAAGCTCTTCTCTTTCAAGAACACTT
GAATTTGCGTTAGACGTTCAGCGATTGCTTCATCAACCGGTTGAATTTCAAGAAGATCAACA
AGCTCTAAACCAGACAACACGTCTTCAGACAGTTTGTCGCCACGTTTAGTGGTACCGCCAC
CATTCGACTCTTGACCGTTCAACAAACGAATTACACGTTCACGTGCTGCTTCTTCAAAGATC
TTGAACTCTTCTTTNNNNNNNNNNNNNNNNNNNNNNNNNGTNNNNNNNTGTNGANTGATNNGA
TNGCCNNNCNTCACNNNNACANNNGTGTNGNN

>T10_2
GGNAAANNNNGNNTTANNNNAANNANNTNNNNGAAGNTTTTCCCACNANCCCNNNNNNNC
GTGACGACCGGCCATCTTATCACCCGGCTGGATGCGACGTTTTACCGCAAGATAAACTTTA
ACTNACTTTCAATACGCCAGTGGTCAGCTCATCGCCTGTAGAAAGTTTGCGTTTTTTCTCAG
CAAATTTCTCGTCAATTTCGAAGCTCTTCTCTTTCAAGAACACTTGAATTTGCGTTAGACGTT

CAGCGATTGCTTCATCAACCGGTTGAATTTCAAGAAGATCAACAAGCTCTAAACCAGACAA
CACGTCTTCAGACAGTTTGTCGCCACGTTTAGTGGTACCGCCACCATTCGACNTCTTGACC
GTTCAACAAACGAAWTACACGTTCACGTGCTGCTTCTTCAAAGATCTTGAACTCTTCTTTNA
NNNNNNTACNNNNNNNNNAGGGTGNNNNNNTGTTGANTGATAANATGGCCNNTCNTCACNN
NAACNNNNNTGTNGANNN

>D13_1
CCGTGACGACCCGCCATCTTATCACCCGGTTGGATGCGACGTTTAACAGCCAAGTAAACCT
TAACAACTTTCAATACACCTGTTGTTAATTCATCACCTGTAGAAAGTTTGCGTTTTTTCTCTG
CAAATTTCTCATCAATTTCGAAGCTCTTCTCTTTCAAGAACACTTGAATTTGAGTTAAACGCT
CTGCAATAGCTTCATCAGTCGGTTGGATTTCAAGTAAATCAACCAACTCTAAACCAGACAAT
ACGTCTGCAGAAAGCTTATCGCCACGCTTAGTTGTACCACCACCGTTAGACTCTTGACCTG
TCAACAAACGAACAATACGTTCACGTGCTGCTTCTTCGAAGATTTTGTATTCTTCTTTNNNN
NNNTTTACNNNAAN

>D13_2
GGATGCGANGTTTAACANGNCCAAGTAAACCNTTAACAACTNTTCAATACACCTGTTGTTAA
TTCATCACCTGTAGAAAGTTTGCGTTTTTTCTCTGCAAATTTCTCATCAATTTCGAAGCTCTT
CTCTTTCAAGAACACTTGAATTTGAGTTAAACGCTCTGCAATAGCTTCATCAGTCGGTTGGA
TTTCAAGTAAATCAACCAACTCTAAACCAGACAATACGTCTGCAGAAAGCTTATCGCCACGC
TTAGTTGTACCACCACCGTTAGACTCTTGACCTGTCAACAAACGAACAATACGTTCACGTG
CTGCTTCTTCGAAGATTTTGTATTCTTCTTTNANNNNNTTACNATAANANNNNNNNNNNNTG
TTGANNNNCTNNNCCCCCCCCCNNNCNNNNNNNNNN

>D16
TCNNCNCCCCTNNNNNNNCGTGACGACCCGCCATCTTATCACCCGGCTGGATGCGACGTT
TCACAGCAAGATAAACTTTAACTACTTTCAATACGCCAGTCGTCAGTTCATCGCCTGTAGAA
AGTTTGCGTTTTTTCTCAGCAAATTTCTCATCAATTTCGAAGCTCTTCTCTTTCAAGAACACT
TGAATTTGAGTTAAACGTTCAGCAATTGCTTCGTCAACTGGTTGAATTTCAAGAAGATCAAC
AAGCTCTAAACCAGACAACACGTCTTCAGACAGTTTGTCGCCACGTTTAGTTGTACCGCCA
CCATTCGACTCTTGGCCGTTGAGTAGACGGATTACACGTTCACGTGCAGCTTCTTCGAAGA
TTTTGAATTCTTCTTTNNNNNNNNTTTNNNANANN
