# Supplementary material for: Multidrug Resistant Acinetobacter Isolates Release Resistance Determinants Through Contact-Dependent Killing and Bacteriophage Lysis
Source: Front Microbiol. 2020 Aug 14;11:1918. doi: 10.3389/fmicb.2020.01918 (PMC7456956; doi:10.3389/fmicb.2020.01918)
Supplement: Supplementary file 2 [file Table_1.DOCX]

**Multidrug resistant *Acinetobacter* isolates release resistance determinants through contact-dependent killing and bacteriophage lysis**

Clay S. Crippen^1^, Michael J. Rothrock Jr.^2^, Susan Sanchez^3^ and Christine M. Szymanski^1^*

^1^Department of Microbiology and Complex Carbohydrate Research Center, University of Georgia, Athens, GA, USA

^2^Agricultural Research Service, United States Department of Agriculture, US National Poultry Research Center, Athens, GA, USA

^3^Department of Infectious Diseases and Athens Veterinary Diagnostic Lab, University of Georgia, Athens, GA, USA

**Contained:**

**Supplementary Figures**


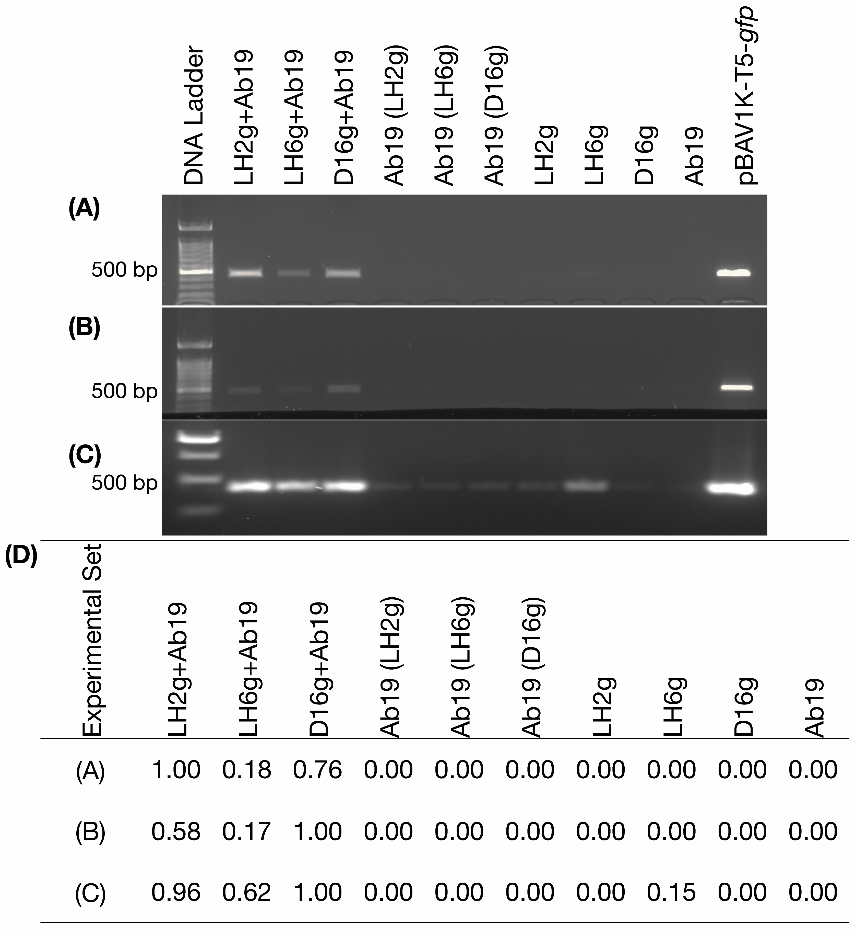


**Figure S1.** Contact-dependent DNA release shown in biological triplicates. (**A-C)** A 1.0% agarose gel depicting the PCR-based KmR gene (450 bp) detection of contact dependent-mediated released DNA. Co-cultures separated by a membrane to inhibit contact are denoted with parentheses. The strains used are as follows: *A. johnsonii* (LH2); *A. radioresistens* (LH6); *A. lwoffii* (D16); and *A. baumannii* ATCC 19606 (Ab19). All strains containing the plasmid pBAV1K-T5-*gfp* are denoted with a “g”. **(D)** Relative intensities of PCR products in each lane from **A-C**, as measured by densitometry using Image Lab™ (Bio-Rad). The brightest band in each experimental group was used as the standard (Rel. Intensity=1.00). Figure S1A is shown in Figure 2C.


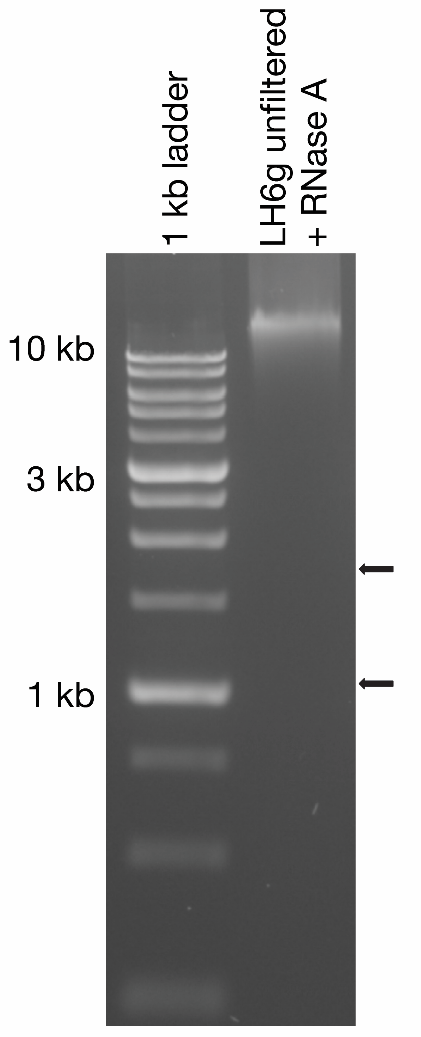


**Figure S2.** RNase A treated *A. radioresistens* “LH6g-unfiltered” seen in Figure 3A. The sample was treated with RNase A to remove host RNA. Arrows indicate the absence of host RNA co-isolated with the host DNA, which are visible in Figure 3A.

**
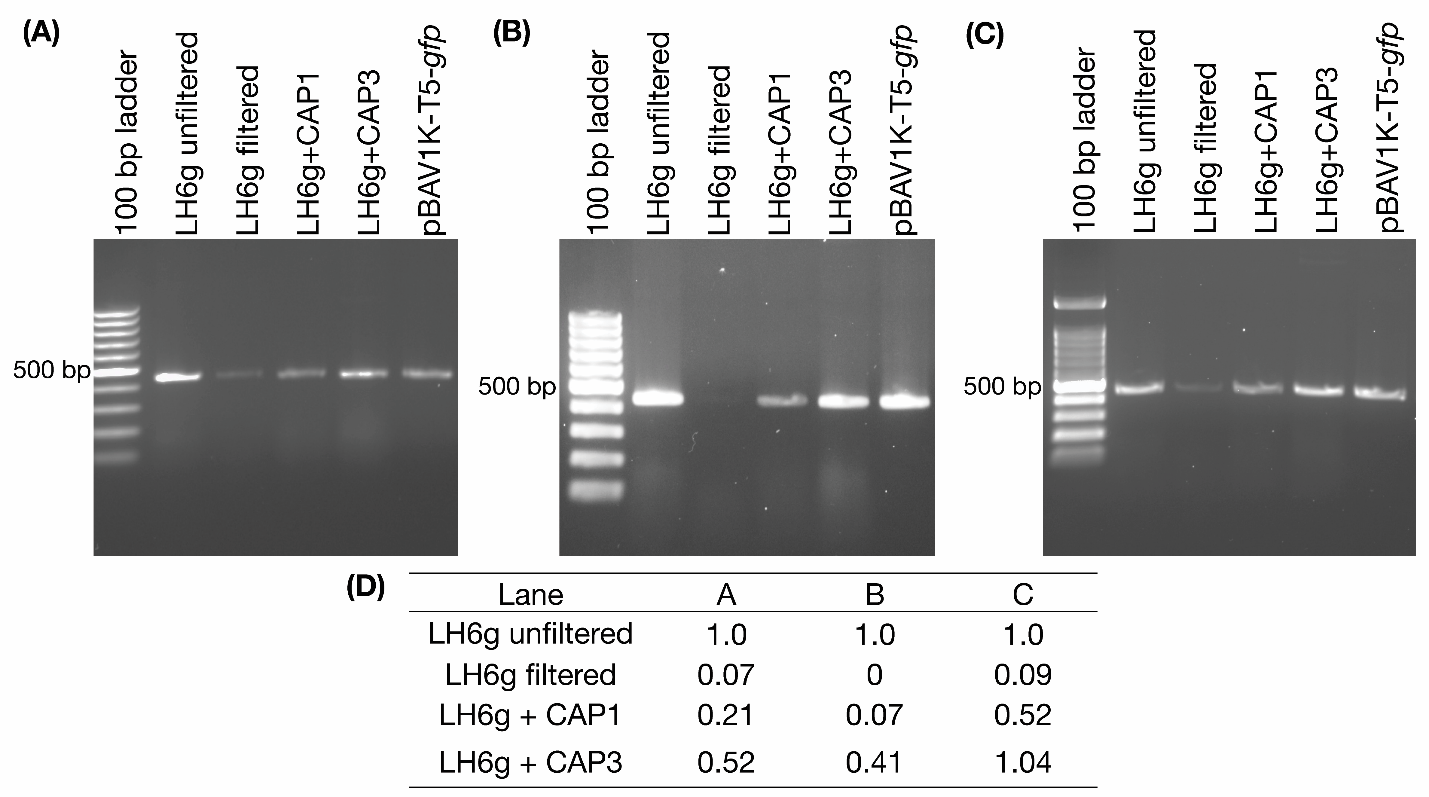
**

**Figure S3.** KmR gene PCR detection in biological triplicates post phage-mediated DNA release. **(A-C)** A 1.0% agarose gel depicting the PCR-based KmR gene (450 bp) detection of bacteriophage-released DNA from the host strain *A. radioresistens* LH6. The “g” designation indicates the presence of pBAV1K-T5-*gfp*. **(D)** Relative intensities of PCR products in each lane from **A-C**, as measured by densitometry using Image Lab™ (Bio-Rad). The bands produced by amplifying KmR gene in LH6g unfiltered samples were used as the standard (Rel. Intensity = 1.0).

**
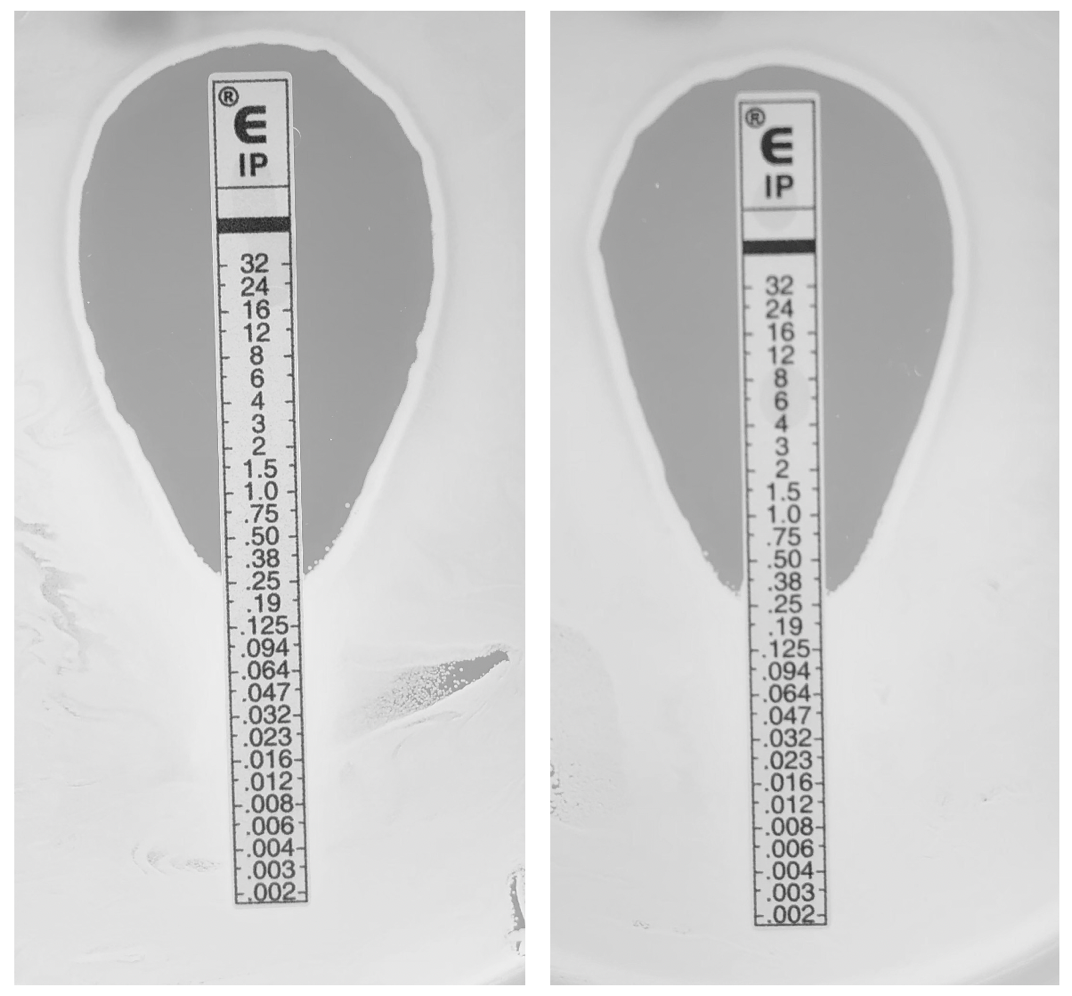
**

**Figure S4.** Imipenem ETEST^®^ strip susceptibility test of *A. radioresistens* LH6 performed on Mueller-Hinton agar. The susceptibility assay, performed in technical duplicate, indicates LH6 is susceptible to imipenem with an MIC of approximately 0.25 µg/mL.
